# Efficacy of Tuina for Myopia in Children: Protocol for a Randomized Controlled Trial

**DOI:** 10.2196/79324

**Published:** 2026-02-12

**Authors:** Shouyao Zhang, Yanju Han, Lijuan Zhang, Fang Tan, Xueping Huang, Can Zhang, Zheng Yao, Lei Xiong, Xiantao Tai

**Affiliations:** 1 The First Clinical Medical College, Yunnan University of Traditional Chinese Medicine, No.1076, Yuhua Road, Chenggong District, Kunming, China; 2 The Second Affiliated Hospital, Yunnan University of Traditional Chinese Medicine, Kunming, China; 3 Department of Tuina, Shiping Hospital of Chinese Medicine, Shiping, China; 4 Department of Tuina, Baoshan Hospital of Chinese Medicine, Baoshan, China; 5 Department of Tuina, Zhaotong Hospital of Chinese Medicine, Zhaotong, China; 6 The Second Clinical Medical College, Yunnan University of Traditional Chinese Medicine, No.1076, Yuhua Road, Chenggong District, Kunming, China, 86 15025189569; 7 The Basic Medical College, Yunnan University of Traditional Chinese Medicine, Kunming, China

**Keywords:** myopia, pediatric tuina therapy, adolescents, randomized controlled trials, protocol, tuina, Tui Na

## Abstract

**Background:**

Myopia has emerged as a major threat to the visual health of adolescents worldwide. Early intervention can effectively slow down the progression of myopia in adolescents. *Tuina* (also known as Tui Na), a significant therapeutic method in traditional Chinese medicine, has shown promising clinical efficacy in delaying the progression of myopia; however, it lacks robust, large-scale, and standardized randomized controlled trials.

**Objective:**

This study aims to explore the efficacy and safety of *tuina* therapy in managing myopia in adolescents, thereby providing solid evidence for the application of *tuina* in the clinical treatment of myopia.

**Methods:**

This study is a multicenter randomized controlled clinical trial. A total of 62 children with myopia will be recruited from 4 hospitals and randomly assigned in a 1:1 ratio to a *tuina* experimental group and a drug-positive control group (tropicamide eye drops). Treatments in each group will be administered 3 times per week for a total of 8 weeks. The *tuina* experimental group will receive 20 minutes of *tuina* therapy per session, while the drug-positive control group will receive tropicamide eye drops administered every other day, with 2 drops per session. The primary outcome measures include uncorrected visual acuity and axial length, while secondary outcome measures include refractive power and accommodative amplitude. Data will be collected at baseline (week 0), on the day of completion of weeks 4 and 8 of treatment, and at the end of the 10-week follow-up period. Adverse events will be monitored and recorded throughout the study. Statisticians will be blinded. Data will be analyzed using SPSS version 28.0.

**Results:**

This study has been funded, and recruitment began in June 2025. As of December 2025, 29 participants have been enrolled, with 16 allocated to the *tuina* group and 13 to the drug-positive control group. Recruitment is expected to continue until October 2026. Final manuscript submission is anticipated by December 2026.

**Conclusions:**

This study aims to evaluate the efficacy and safety of *tuina* therapy in the treatment of adolescents with myopia. We hypothesize that the therapeutic effect of *tuina* therapy is noninferior to that of tropicamide eye drops, with the additional advantages of fewer side effects and stable long-term efficacy, thereby providing reliable evidence and support for the application of *tuina* therapy in the management of myopia in adolescents.

## Introduction

### Background

Myopia, a common ocular disorder predominantly developing during childhood and adolescence, is characterized by excessive elongation of the eye globe, leading to the focusing of distant object images in front of the retina and thereby causing blurred distance vision. With continuous advancement of socioeconomic levels [1], the widespread prevalence of mobile electronic devices [2], and a lack of outdoor activities [3], the problem of myopia among adolescents has become increasingly severe. Currently, the global population with myopia is approximately 2 billion individuals (28.3% of the global population), and it is estimated that by 2050, the prevalence of myopia will increase to 4.76 billion individuals, approximately half of the global population [4]. Among school-aged children (6‐19 y old), the prevalence of myopia in East Asian regions is the highest (73%), which is significantly higher than that in other regions of the world, including North America (42%), Europe (40%), South America (10%), and Africa (3.4%‐4%) [5]. Taking China as an example, more than 80% of high school students suffer from myopia [6], which not only has a significant impact on children’s academic performance and future career choices [7] but also serves as a major cause of incurable vision impairments, such as glaucoma, retinal detachment, and macular degeneration [8]. Owing to the extremely high prevalence rate, myopia has caused tremendous losses to socioeconomic development. A research report stated that in 2019, productivity loss caused by severe visual impairment and blindness was approximately US $94.5 billion, and it is expected to rise to US $229.3 billion by 2050 [9]. Therefore, researchers and clinicians are eager for effective treatment methods that can delay or even prevent the progression of myopia in children.

Currently, common interventions for myopia include surgical and nonsurgical methods. Refractive surgery is used to correct the vision; however, high costs [10] and postoperative complications, such as dry eye syndrome, are drawbacks that are difficult to ignore in surgical treatments [11]. Nonsurgical interventions include pharmacological and nonpharmacological treatments, with pharmacological therapy commonly using low-concentration atropine; however, photophobia [12] and dry eye syndrome are common complications. Tropicamide eye drops are a cholinergic receptor antagonist medication. Compared with atropine, tropicamide has the advantages of rapid metabolism, fewer adverse reactions, higher safety, and higher patient compliance and adherence. Evidence suggests that, compared with atropine treatment, tropicamide eye drops can improve the clinical treatment outcomes and enhance the quality of life in adolescents with myopia [13].

Optical therapies, such as myopia-control glasses, including highly aspheric lenslets, spectacle lenses with slightly aspheric lenslets, and single-vision spectacle lenses, are widely applied methods for myopia control and can effectively slow the progression of myopia and axial elongation [14]. However, a large-scale network meta-analysis indicated that the GRADE (Grading of Recommendations Assessment, Development, and Evaluation) evidence level for myopia-control spectacles is relatively low, and their true efficacy requires further investigation [15]. Corneal reshaping lenses can correct myopia to some extent but are associated with adverse reactions, such as corneal infections [16], and many children find these lenses difficult to tolerate. Some studies have shown that more than half of children were unable to complete the treatment [17]. Moreover, high costs necessitate long-term consideration when choosing contact lenses. A study on the cost of myopia treatment over 5 years found that corneal reshaping lenses were the most expensive, with an approximate cost of US $15,347 [10]. Recent research has found that repetitive low-intensity red light therapy can help improve axial length (AL), control vision in children with high myopia, and increase macular choroidal thickness [18]; however, there is still a certain rebound effect after the cessation of repetitive low-intensity red light treatment [19].

*Tuina* (also known as Tui Na), one of the nonpharmaceutical therapies characteristic of traditional Chinese medicine (TCM), can be traced back to the Qin and Han dynasties (99 BC-26 BC) [20,21] and has a history of more than 2000 years. Massage, acupressure, acupoint eye exercises, and other techniques all fall under the category of *tuina*. Existing research has demonstrated that localized *tuina* therapy around the eyes can promote meridian circulation, enhance blood circulation, and alleviate spasms of the ciliary and extraocular muscles [22-24]. These effects may be associated with improvements in retinal thickness, choroidal thickness, perfusion area, and vascular density through acupoint massage around the eyes [25]. Tian et al [26] evaluated the impact of eye exercises on AL in adolescents and found that the frequency of eye exercises was conducive to controlling the trend of axial elongation. Moreover, a large-scale meta-analysis suggested that acupoint stimulation around the eyes has a moderate protective effect in controlling myopia; however, incorrect execution and attitudes may have a negative impact on vision health [27]. This indicates that the correct method of stimulating ocular acupoints is of paramount importance for the recovery of vision.

The holistic concept of TCM emphasizes the dialectical relationship between local functions and the whole body, with the various parts of the human body being inseparable in structure and mutually influential in function. Normal vision is a result of the proper functioning of all organs. A sole focus on local manipulation neglects the holistic perspective of TCM, potentially affecting the final clinical efficacy. Therefore, we have developed an integrative approach combining overall regulation with local treatment, known as selective spinal *tuina* manipulation therapy, as a potential intervention for myopia in adolescents. However, high-quality medical evidence supporting this approach is currently lacking.

### Objectives

This study aims to conduct a high-quality randomized controlled trial to objectively evaluate the efficacy and safety of selective spinal *tuina* manipulation therapy in the treatment of childhood myopia. We hypothesize that selective spinal manipulation therapy can improve vision in children with myopia and is safer than drug therapy, with no side effects and more stable long-term efficacy.

## Methods

### Study Design

**Figure 1.**
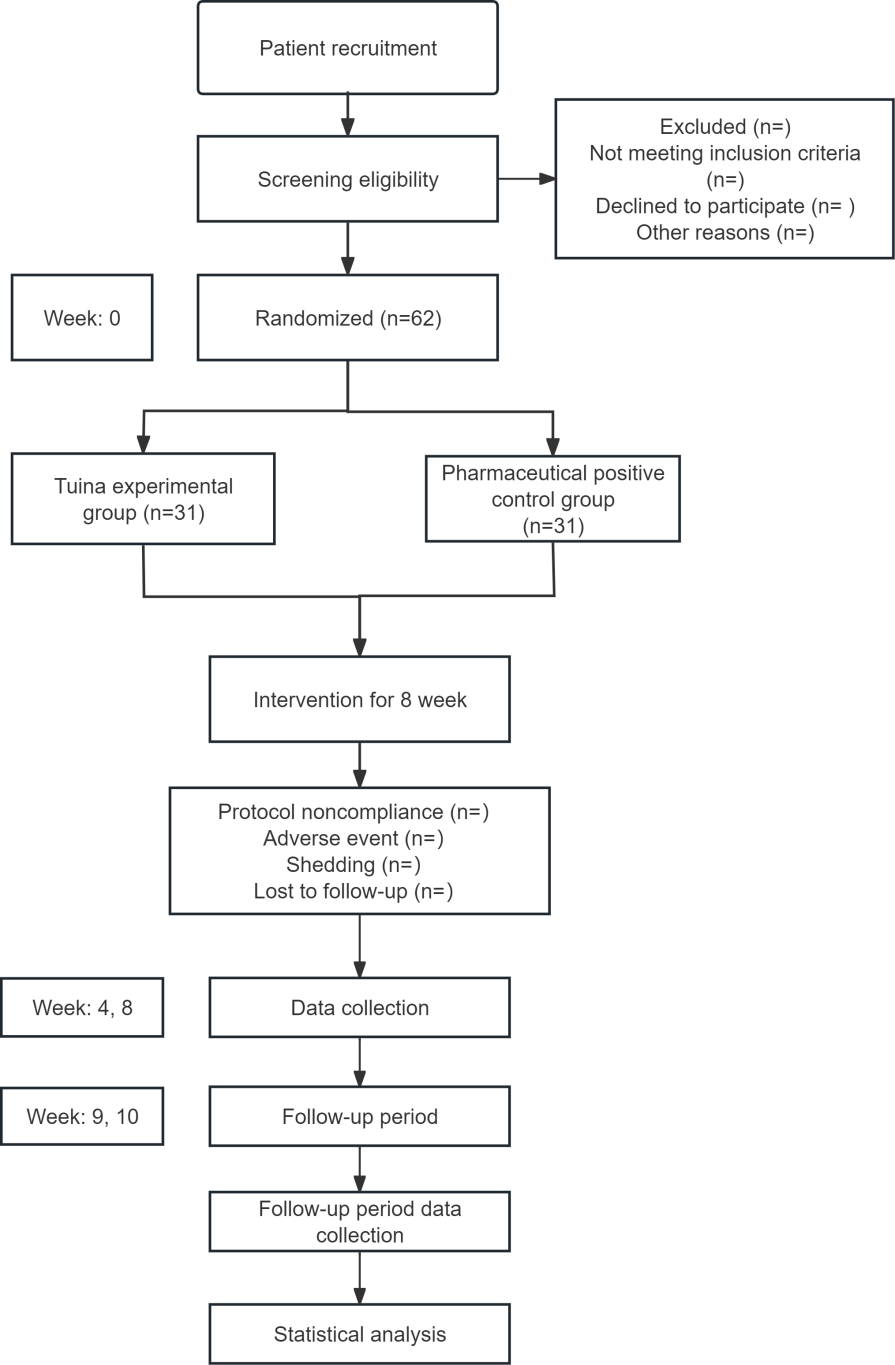
Flowchart of this study.

This study is designed as a multicenter randomized controlled noninferiority trial to be conducted from June 2025 to October 2026. We plan to recruit 62 participants from 4 hospitals in Yunnan Province. Eligible participants will be randomly assigned in a 1:1 ratio to the *tuina* experimental group and the positive drug control group (tropicamide eye drops). Each participant will undergo an 8-week treatment period followed by a 2-week follow-up. Baseline information and outcomes will be assessed at baseline, at the completion of weeks 4 and 8 of treatment, and at the end of the 10-week follow-up (Figure 1). This trial adheres to the guidelines of the SPIRIT (Standard Protocol Items: Recommendations for Interventional Trials).

### Participants

#### Recruitment Strategy

This study will recruit 62 participants through recruitment notices in communities and hospitals from the Second Affiliated Hospital of Yunnan University of Traditional Chinese Medicine, Baoshan Hospital of Chinese Medicine, Shiping Hospital of Chinese Medicine, and Zhaotong Hospital of Chinese Medicine. Recruitment will be conducted by physicians from the *tuina* departments of hospitals. Appropriate compensation will be provided to participants upon completion of the entire procedure. Moreover, the parents or legal guardians of the participants have the right to withdraw from this study at any time and for any reason without facing discrimination or difficulties in the hospital. Participants must be children with myopia and must meet the following inclusion criteria.

#### Diagnostic Criteria

Myopia will be diagnosed according to the standards outlined in the White Paper on Myopia Control and Prevention Research established by the International Myopia Institute under the World Health Organization [28,29], as well as the “Diagnostic and Therapeutic Criteria for Internal Medicine Diseases in Traditional Chinese Medicine” (ZY/T001.1‐94), a standard of the People’s Republic of China for Traditional Chinese Medicine [30].

1. Mild myopia: −0.50D to −3.00D
2. Moderate myopia: −3.00D to −6.00D
3. High myopia: above −6.00D

#### Inclusion Criteria

The inclusion criteria are as follows:

1. Children who undergo routine ophthalmic examination before enrollment and meet the diagnostic criteria for simple mild-to-moderate myopia in both TCM and Western medicine
2. Age range: 6 to 12 years, gender-neutral
3. Guardians and children who sign the informed consent forms
4. Guardians and children who volunteer to participate in clinical observation, comply with the physician’s arrangements, and cooperate with treatment

#### Exclusion Criteria

The exclusion criteria are as follows:

1. Presence of ocular inflammation, facial dermatitis, or infectious lesions
2. Patients with severe systemic diseases, such as cardiovascular, cerebrovascular, hepatic, renal, hematological, and psychiatric disorders
3. Individuals with a history of congenital hereditary high myopia
4. Patients with pathological myopia fundus changes and/or significant visual impairment, or other concomitant ophthalmic diseases that may affect the assessment of efficacy
5. Children suffering from neurological and psychiatric disorders (eg, cerebral palsy, autism, spina bifida, anorexia nervosa)

### Sample Size

Using G*Power software to calculate the sample size, based on the preliminary pilot study results, the expected efficacy rate in the manipulation treatment group is above 80% (*P*<.05). Therefore, we set an expected effect size of 0.8. To detect between-group differences with 80% statistical power (*β*=.20), we choose a 2-tailed α level of .05. With an allocation ratio of 1:1, the required sample size was 52 participants (26 per group). Considering a 20% expected dropout rate, the final sample size was set at 62 participants (31 per group).

### Randomization and Blinding

The head of each hospital and the relevant department will recruit participants. Eligible children with myopia will be randomly assigned on a 1:1 basis to either a *tuina* therapy intervention group or a drug positive control group. Randomization will be conducted by the head of each hospital using a computer-generated random number generator. Upon the entry of qualified trial participants, the hospital head will assign them to the respective groups according to the randomly generated numbers and follow the corresponding medical instructions without any modification. There are 2 types of interventions in this study. It is difficult to implement blinding for both the therapists and the parents or legal guardians of the children. Nevertheless, the outcome assessors will use blinding for the randomization, intervention procedures, and result analyses.

### Intervention

#### Selection of Therapists and Participants

To ensure that all *tuina* manipulation in this study conforms to standardized procedures, the therapists involved possess physician qualifications and a master’s degree in pediatric *tuina* and have undergone unified training in the *tuina* techniques used in this study. Only those who successfully complete the training are eligible to participate. During the course of the experiment, children with myopia are generally not allowed to use any other interventions. If participants adopt other interventions during this period, they will be defined as noncompliant and withdrawn from the experiment.

#### *Tuina* Experimental Group

Participants will receive 20-minute pediatric *tuina* treatments. The treatments will be administered 3 times a week for a total of 8 weeks. A professor specializing in pediatric *tuina* will determine the specific techniques. The techniques will require a light and gentle touch, steady and substantial. Children will be placed in a quiet and warm treatment room with a temperature maintained between 25 °C and 28 °C, ensuring they are in a state of calm. The specific steps are as follows: (1) Step 1: holistic regulation; (2) Step 2: selective stimulation of back-sensitized acupoints; (3) Step 3: symptomatic treatment; and (4) Step 4: harmonization of Yin and Yang.

**Figure 2.**
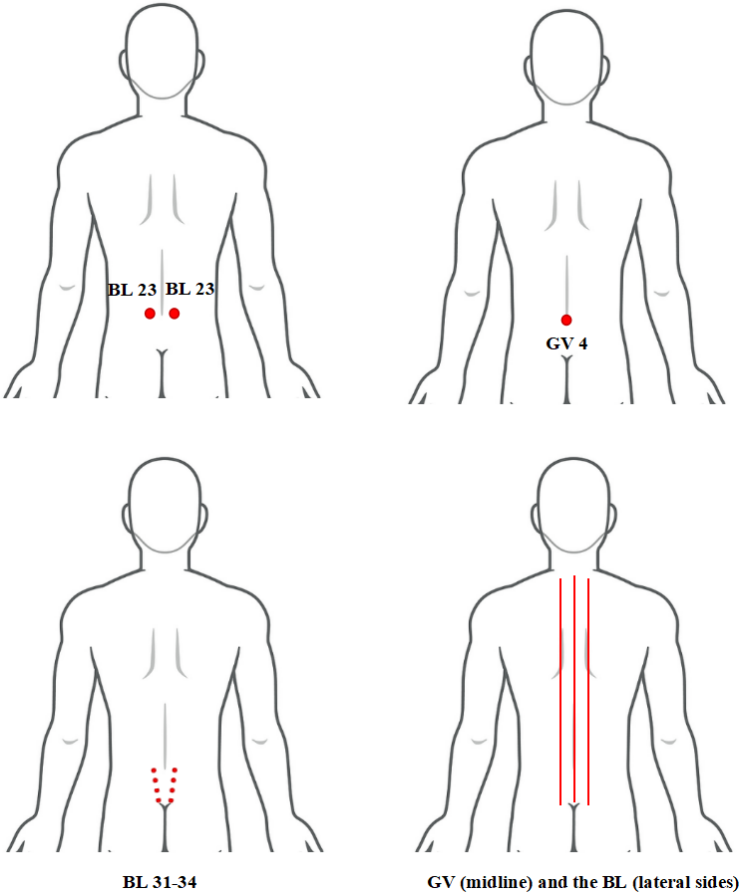
Holistic regulation. Step 1 of selected spinal manipulative therapy. BL: Bladder Meridian of Foot-Taiyang; GV: Governor Vessel.

First, based on the holistic concept and the principle of adhering to the disease mechanism, we will apply *tuina* to the Governor Vessel (GV) and the Bladder Meridian of Foot-Taiyang (BL):

- The child will be placed in a prone position, with the practitioner standing on the right side of the child. They will use the thumb to knead the entire spine in the direction of the GV from top to bottom, 3 times for 1 minute.
- The practitioner will knead the first and second lateral lines of the bladder meridian from top to bottom using the index and middle fingers, 3 times for 1 minute.
- The practitioner will use the thumb and index finger to pinch and lift the skin of the spinal column surface, performing this action from bottom to top 3 times in 1 minute.
- The practitioner will rub the acupoints of BL 23, GV 4, and BL 31‐34, using the palm or the thenar eminence to firmly press against the skin and perform rapid linear reciprocating rubbing movements for 1 minute (Figure 2).

Second, the child will remain in a prone position, and the practitioner will stand on the right side. The practitioner will perform palpation of the cervical and dorsal regions to identify positive reaction points (linear or nodular reaction points). We will confirm the sensitized acupoints using infrared thermography. The practitioner will perform targeted stimulation using kneading manipulation for 2 minutes (Multimedia Appendix 1).

**Figure 3.**
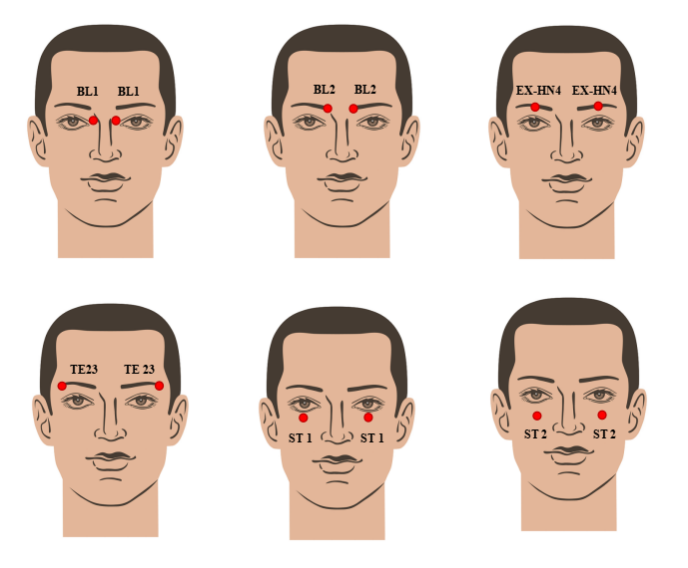
Symptomatic treatment. Step 3 of selected spinal manipulative therapy. BL: Bladder Meridian of Foot-Taiyang; EX-HN: Eight Extraordinary Meridians-Head and Neck; ST: Stomach Meridian of Foot-Yangming; TE: Triple Energizer Meridian of Hand-Shaoyang.

Third, for symptoms of dry eyes and blurred vision, the practitioner will apply the kneading technique to BL 1, BL 2, Eight Extraordinary Meridians-Head and Neck (EX-HN) 4, Triple Energizer Meridian of Hand-Shaoyang (TE) 23, Stomach Meridian of Foot-Yangming (ST) 1, and ST 2, with each acupoint treated for 1 minute, totaling 6 minutes. They will gently rub both palms together to generate heat and then apply the warmth to the eyes for 1 minute (Figure 3).

**Figure 4.**
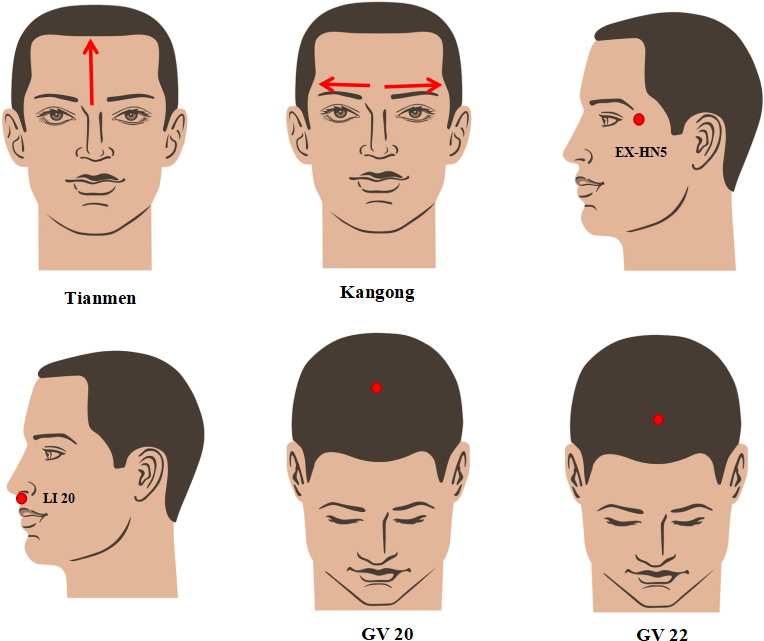
Harmonization of Yin and Yang. Step 4 of selected spinal manipulative therapy. EX-HN: Eight Extraordinary Meridians-Head and Neck; GV: Governor Vessel; LI: Large Intestine Meridian of Hand-Yangming.

Fourth, the child will assume a supine position, and the practitioner will stand on the side of the child’s head. The practitioner will alternately push the “Tianmen” and the “Kangong” with both thumbs 50 times for each and knead EX-HN5 for 1 minute with the middle fingers, totaling 2 minutes. The practitioner then will use the thumbs to knead Large Intestine Meridian of Hand-Yangming (LI) 20 and the palm to rub GV 22 and GV 20 circularly for 1 minute each, totaling 3 minutes. We will then place the patient in a sitting position, and the practitioner will lightly pat the back with a hollow palm, performing this 3 times from top to bottom, for 1 minute. The thumb and index finger, along with the middle finger, will pinch the BL 17 3 times for 1 minute (Figure 4).

For the location and manipulation details of each acupoint, see Multimedia Appendix 1.

#### Drug-Positive Control Group

The positive-drug control group will receive treatment with compound tropicamide eye drops (Shenyang Xingqi Co., Ltd.; product specification: 1 ml per vial) at a frequency of once every 2 days before bedtime, with 2 drops per administration. The group will continue the treatment for a duration of 8 weeks.

### Outcomes Measures

#### Primary Outcome

##### Uncorrected Visual Acuity

We use an international standard visual acuity chart to assess visual acuity, record uncorrected visual acuity (UCVA) results in decimal notation, and subsequently convert them into logMAR format for statistical purposes. The complete disappearance of symptoms such as visual fatigue and exotropia, the restoration of distant vision, and the achievement of a visual acuity of 5.0 on the visual acuity chart are indicative of a cure; the basic disappearance of symptoms with an improvement of 3 to 5 lines on the visual acuity chart is indicative of marked efficacy; a significant alleviation of symptoms with an improvement of 2 lines on the visual acuity chart is indicative of efficacy; no change in symptoms with an improvement of 1 line on the visual acuity chart is indicative of inefficacy. The brightness of the visual acuity chart box must meet the latest national standards, which stipulate that the brightness of the visual acuity chart box should not be less than 200 cd/m^2^. We check the brightness of the examination room, as it affects the size of the pupil and use moderate appropriate lighting, with a diameter of the pupil around 3 mm being optimal.

##### Axial Length

We measure AL using ophthalmic A-ultrasound, obtain 5 readings with differences between them all less than 0.05 mm, and take the average. Normal AL for adolescents ranges from 22 to 24 mm, and values outside this range represent refractive errors.

### Secondary Measures

#### Spherical Equivalent

One drop of 1% cyclopentolate is instilled at 0, 5, and 20 minutes to induce cycloplegia in both eyes. Fifteen minutes after instillation, cycloplegia affects the pupillary light reflex and the size of the pupil. Automatic cycloplegic refraction is performed using an automatic refractor (KR-8800, Topcon). Three readings are obtained for each eye, and the average is calculated until the desired precision (spherical and cylindrical refractive power of 0.25 D and axis of 5°) is achieved. We calculate the spherical equivalent (SE) refraction as the sum of the spherical value and half the cylindrical value.

#### Amplitude of Accommodation

**Figure 5.**
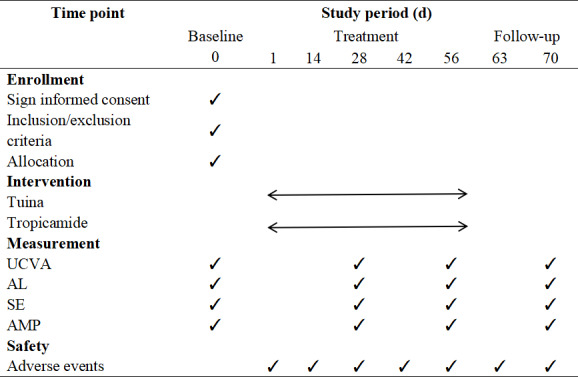
Study schedule of measurements at different time points. AL: axial length; AMP: amplitude of accommodation; SE: spherical equivalent; UCVA: uncorrected visual acuity.

We determine the ability of the lens to change refractive power in response to near stimuli using the negative lens method. Refractive status is corrected using a comprehensive refractor (RK-F2, Canon, Japan), with the near visual target fixed at 40 cm. We instruct the participants to fixate on the line above the best vision on the near visual acuity chart and gradually add negative lenses in increments of −0.25 D until the participant experiences persistent blur, at which point we restore the previous degree. The amplitude of accommodation (AMP) is calculated as the total degree of added negative lenses (taking the positive value) plus 2.50 D (Figure 5).

### Data Analysis

All data will be analyzed using SPSS version 28.0 and GraphPad Prism version 10.3.1 statistical software. For normally distributed data, results will be described as mean (SD) and analyzed using the *t* test. For nonnormally distributed data, results will be described as median (IQR) and analyzed using the Wilcoxon rank sum test for within-group comparisons and the Mann-Whitney *U* test for between-group comparisons. A 2-sided *P* value of .05 will be considered statistically significant. Missing data will be handled using expectation maximization, last observation carried forward, or listwise deletion.

### Adverse Events Reporting and Compliance Enhancement

From week 0 to week 10, detailed records of adverse events (AEs) will be documented, including type, occurrence time, severity, and duration. In the event of visual impairment, skin injuries, fainting, infections, or other serious AEs, treatment will be discontinued, and the participant will be transported to the nearest hospital. A detailed AE report will be submitted to the Ethics Committee. At the conclusion of the follow-up period, children are entitled to receive 3 sessions of pediatric *tuina* therapy at no cost; however, those who withdraw prematurely are not eligible for this benefit.

### Data Management and Quality Control

In this study, the basic information of each participant includes gender, age, height, weight, medical history, current condition, and contact information for parents or legal guardians. Researchers promptly complete the case report form (CRF) for each participant, including UCVA, SE, AL, AMP, concomitant medications, dropout status, and AEs (such as blurred vision, photophobia, etc). Regardless of whether these AEs are related to the treatment of this study, participants will receive appropriate management and timely documentation, which we record in detail in the CRF. Four rounds of outcome data are collected from baseline to the end of follow-up. Completed CRF forms from 4 hospitals are sent to the Department of Tuina Basic Education of the Second Clinical Medical College of Yunnan University of Chinese Medicine. Two members who are unaware of the grouping status will conduct double data entry and aggregate the data into an electronic dataset. Simultaneously, the data administrator monitors and checks for any procedural errors. Unblinding is permissible after the completion of the experiment. The Data Monitoring Committee is independent of the sponsor and has no conflicts of interest.

### Ethical Considerations

The trial has been registered on the Chinese Clinical Trial Registry (ChiCTR2600116606). At the onset of this study, we submitted an application to the Chinese Clinical Trial Registry Center; however, the review process took an extended period of time, resulting in registration occurring only after the recruitment and data collection had commenced. This study was approved by the Ethics Committee of the Second Affiliated Hospital of Yunnan University of Chinese Medicine (2024‐050) and conforms to the Declaration of Helsinki. It will be ensured that participants actively provide informed consent or discuss and make decisions with trusted family members. All participants’ personal identification information, including names, telephone numbers, identification numbers, and medical records, is anonymized to prevent information leakage. Researchers store all participants’ data in a dedicated cabinet and retain them for at least 5 years after publication. The Ethics Committee of Yunnan University of Chinese Medicine will regularly review the progress of the trial and supervise the collection, distribution, and confidentiality of the data. The committee can implement modifications or termination of the trial. The use of all personal photographs has been approved by the individuals concerned and their guardians, and informed consent forms have been signed (Multimedia Appendix 2). Upon completion of the treatment and follow-up, the participants will be provided with 3 iterations of *tuina* therapy at no cost. Participants who withdraw before completion will not be eligible for this benefit. All treatments and tests received by participants are free of charge. Participants will be allowed to withdraw at any time, and their subsequent treatment will not be affected in any way.

## Results

As of December 2025, 29 participants have been enrolled, with 16 allocated to the *tuina* experimental group and 13 allocated to the drug-positive control group. The current data indicate that *tuina* therapy is helpful in enhancing UCVA and improving AL of eyes, with no AEs reported, suggesting that *tuina* therapy is an effective alternative treatment. The enrollment process will continue until October 2026. We will submit the final manuscript before December 2026, following which we will disseminate the final results in accordance with the CONSORT checklist.

## Discussion

### Expected Findings

This study aims to evaluate the efficacy and safety of *tuina* therapy through a randomized controlled clinical trial. We anticipate that children who receive *tuina* therapy will show improvements in AL, UCVA, SE, and AMP that are not inferior to those achieved through drug therapy. Furthermore, we expect that *tuina* therapy will be safer, free of adverse effects, and exhibit more stable long-term efficacy.

The high prevalence and severe complications of myopia have inflicted significant damage on the visual health of adolescents, imposing a substantial burden on both society and the economy. Therefore, the urgent need for an effective method to prevent and control myopia is evident. *Tuina* is a potential alternative therapy for myopia. However, current research on the application of *tuina* therapy for myopia predominantly focuses on local ocular areas, neglecting the holistic perspective, which to some extent limits its therapeutic efficacy. The holistic perspective is a fundamental concept in TCM, which views the human body as an integrated whole. This perspective posits that the human body is a harmonious entity, with the heart as the master and the 5 organs at the center, interconnected through the meridian system. This holistic entity is indivisible in terms of structural integration, functionally coordinated, and pathologically interrelated. Consequently, in the rehabilitation of myopia, it is imperative not only to address local symptoms around the eyes but also to promote the overall regulation of the child’s body.

During our long-term clinical practice, we have observed that children with myopia exhibit corresponding abnormalities at specific acupoints on their backs, such as BL 13, BL 15, BL 18, BL 20, BL 23, and their surroundings. These abnormalities include the presence of cord-like or nodular reactive substances, or localized temperature decreases. Selectively stimulating these abnormal reaction points on the back acupoints (or regions) can effectively improve the visual acuity of children. Further research has revealed that the acupoints of the first lateral line of the BL are highly congruent with the dermatomal projections of the spinal nerve roots on the surface of the body. Based on these findings, we have integrated the holistic perspective of TCM with the anatomical principles of spinal nerves in Western medicine, thereby establishing “selective spinal *tuina* manipulation therapy.” This technology system consists of 4 steps: the first and fourth steps focus on overall regulation, while the second and third steps emphasize localized treatment. This approach achieves a combined model of diagnosis and treatment that integrates both global and local care, leading to the restoration of vision.

The first step is comprehensive treatment to achieve overall rehabilitation objectives. The principle underlying this approach is the stimulation of the GV and the Bladder Meridian, which is closely associated with the regulation of visceral functions. According to *《Yin Hai Zhi Nan》*, “In treating the eyes, one cannot afford to overlook the Bladder Meridian.” The Bladder Meridian is the meridian with the highest number of acupoints in the human body. Each internal organ corresponds to a specific acupoint on this meridian. By stimulating the acupoints on this meridian, one can regulate the body’s neural and humoral functions, enhance the body’s *qi*, and achieve the purpose of preventing disease [31]. The second step involves selective stimulation of back sensitized acupoints or regions to achieve personalized and precise rehabilitation objectives. The sensitization of acupoints refers to the transition from a “quiescent” (physiological state) to an “activated” (pathological state) condition, primarily manifesting as abnormalities in morphological, thermal, and electrophysiological indicators. By stimulating abnormal back acupoints, the regulation of visceral functions can be achieved. The third step involves addressing common symptoms associated with myopia, such as blurred vision and visual fatigue, through local massage stimulation around the eye area. This can help to relax muscles, promote blood circulation, and enhance vision. A meta-analysis on the selection rules of acupoints for myopia indicates that local eye acupoints such as BL 1, BL 2, and ST 2, which have branches of the supraorbital nerve, oculomotor nerve, and facial nerve, as well as the orbital vessels and nerves, may promote increased blood flow to the eyes, enhance orbital blood supply, and stimulate orbital nerves to regulate, thereby coordinating the contraction and relaxation of eye muscles and exerting regulatory effects on myopia [32,33]. Fourth, harmonizing *yin* and *yang* involves stimulating acupoints along the GV and related specific points on the head and face, further promoting overall rehabilitation. Stimulating acupoints on the head and face can enhance cognitive function and calm the mind, while rubbing the back along the GV and Bladder Meridian can regulate the flow of *qi* throughout the body, adjusting the functions of the organs. By integrating these methods, the circulation of blood and *qi* can be promoted, and *yin* and *yang* can be harmonized, ultimately achieving the rehabilitation of myopia.

### Innovations and Limitations

It should be noted that this study also has certain limitations: First, given that *tuina* therapy relies on the proficient mastery of techniques and acupoints by the practitioner, it is difficult to implement a blinded design. As a form of physical contact between the practitioner and the patient, *tuina* therapy, whether or not it targets specific acupoints, inevitably stimulates the skin, muscles, and peripheral nerves. Therefore, it is also challenging to blind the patients. Consequently, a sham *tuina* group was not included. However, relatively standardized operational protocols and procedures have been established, and all participating practitioners must strictly adhere to them in an effort to minimize such differences. Nevertheless, completely eliminating such differences is challenging. If subsequent analyses reveal that these differences significantly impact the results, they will be considered incorporating them into our analytical models to ensure the accuracy and reliability of our findings. Second, due to financial and human resource constraints, this study does not conduct a multicenter trial involving the entire country. Future work will aim to expand the scope of the study.

### Conclusions

This study will investigate the efficacy and safety of *tuina* therapy in the prevention and treatment of myopia in adolescents, providing evidence for clinical decision-making and supporting the inheritance and development of *tuina* therapy. It is noteworthy that previous studies have not investigated the intervention effects of *tuina* therapy on myopia from the perspective of TCM holistic philosophy. This study will be the first to explore the effects of *tuina* therapy on myopia in adolescents from the perspective of TCM holistic philosophy. This study could support *tuina* therapy as an effective method for managing myopia in adolescents, offering potential benefits to patients and their caregivers.

## Supplementary material

10.2196/79324 Multimedia Appendix 1 Details of *tuina* manipulation.

10.2196/79324 Multimedia Appendix 2 Informed consent.

## Abbreviations

AE: adverse event
AL: axial length
AMP: amplitude of accommodation
BL: Bladder Meridian of Foot-Taiyang
CRF: case report form
EX-HN: Eight Extraordinary Meridians-Head and Neck
GRADE: Grading of Recommendations Assessment, Development, and Evaluation
GV: Governor Vessel
LI: Large Intestine Meridian of Hand-Yangmin
SE: spherical equivalent
SPIRIT: Standard Protocol Items: Recommendations for Interventional Trials
ST: Stomach Meridian of Foot-Yangming
TCM: traditional Chinese medicine
TE: Triple Energizer Meridian of Hand-Shaoyang
UCVA: uncorrected visual acuity
