# Supplementary material for: Efficacy of Tuina for Myopia in Children: Protocol for a Randomized Controlled Trial
Source: JMIR Res Protoc. 2026 Feb 12;15:e79324. doi: 10.2196/79324 (PMC12900509; doi:10.2196/79324)
Supplement: Multimedia Appendix 1 [file resprot-v15-e79324-s001.docx]

Multimedia Appendix 1 Details of *Tuina* Manipulation

**BL1**

**Location:** It is at the depression 0.1 cun medial to the inner superior corner of the inner canthus of the eye.

**BL2**

**Location:** Extending from the BL1 upward to the edge of the eyebrow, where a depression can be palpated at the frontal cleft.

**EX-HN4**

**Location:** At the forehead, directly above the pupil, between the eyebrows.

**TE23**

**Location:** At the forehead, within the depression at the ends of the eyebrows.

**Manipulation type:** Pressing Manipulation. Using the pad of the thumbs, apply pressure gradually in a downward direction, starting from a light touch and increasing in depth.

**Duration time:**1 minute.

**Intensity:** It is advisable to experience a local sensation of soreness and distension.


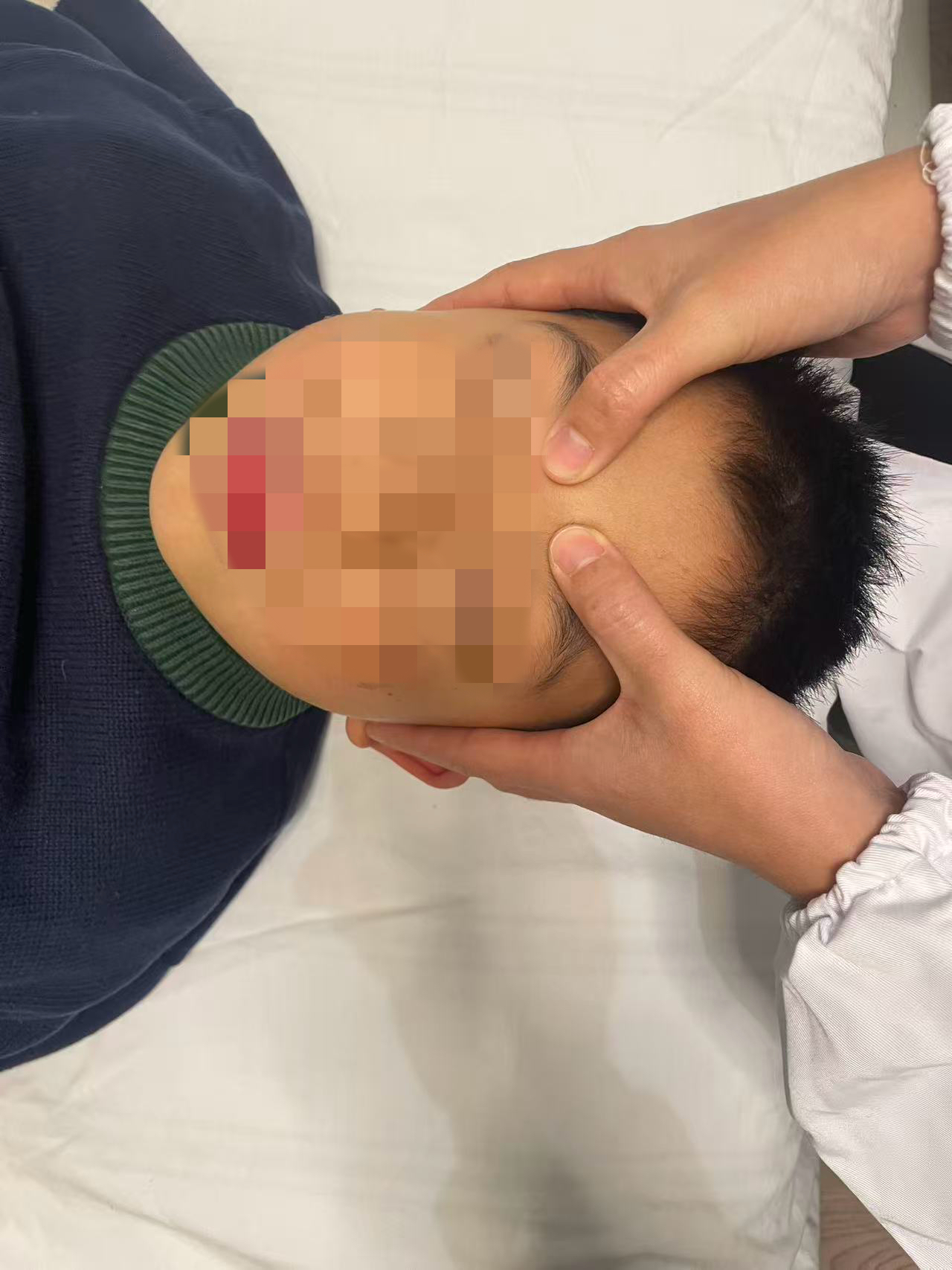

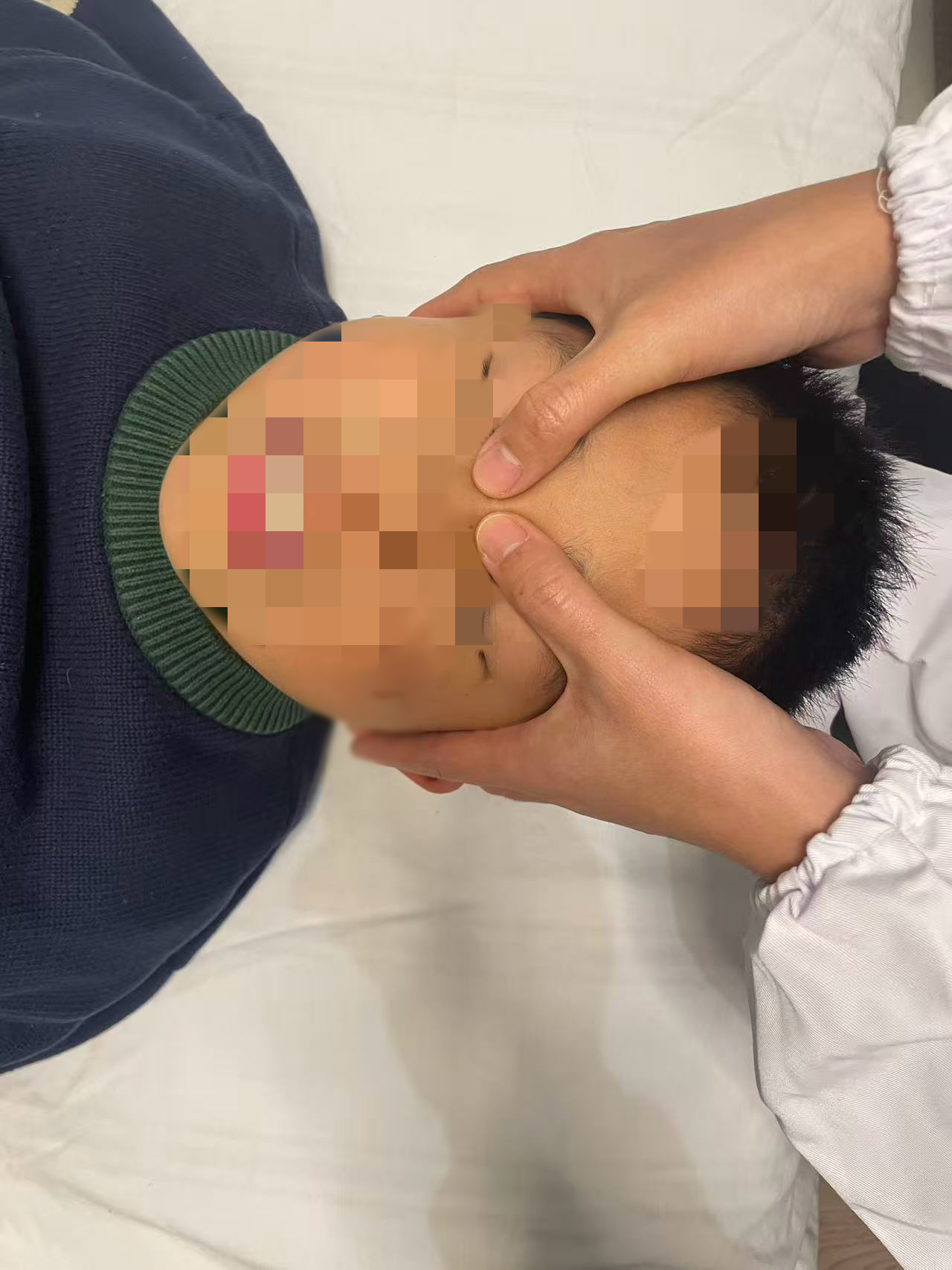

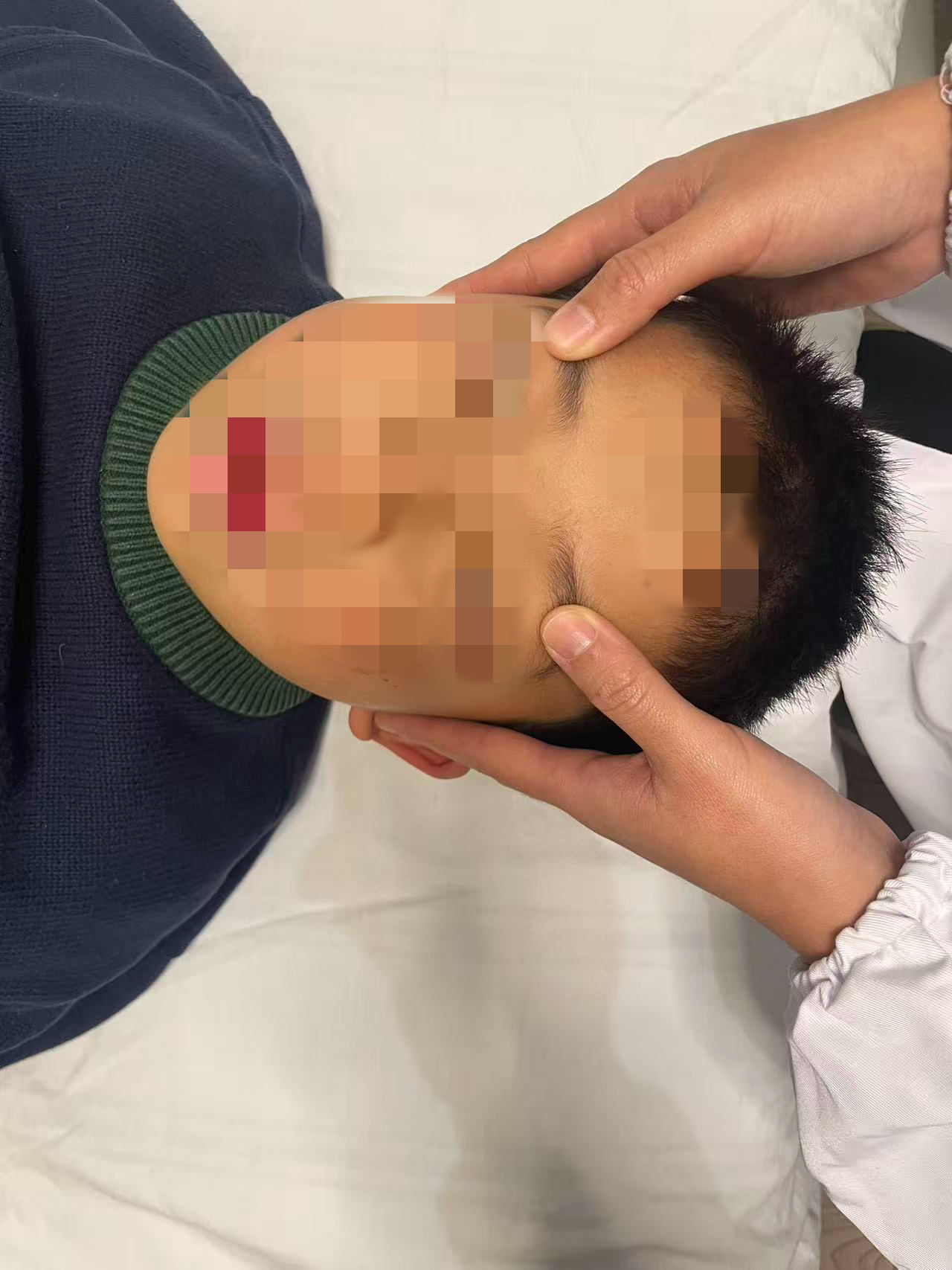

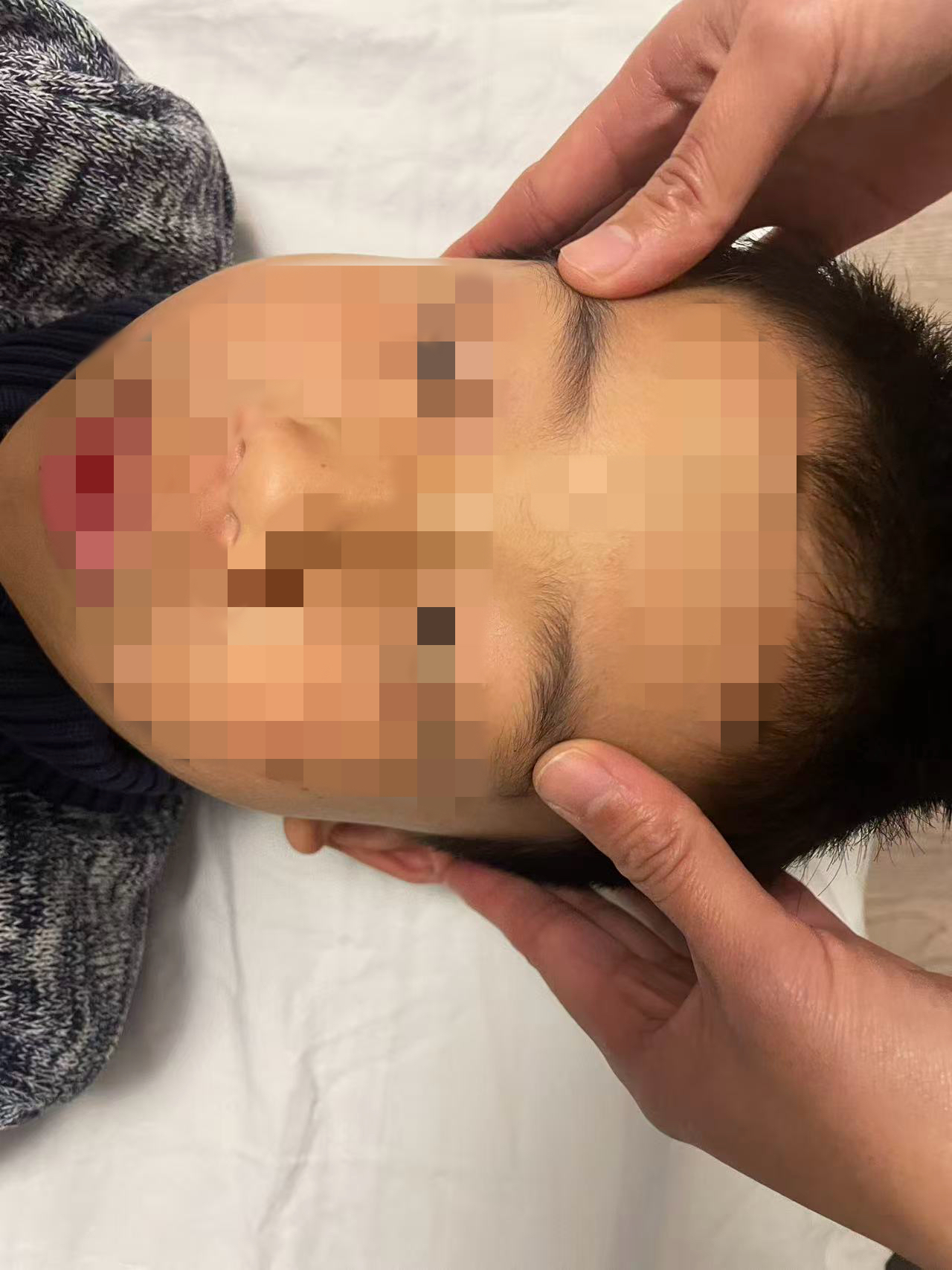


**ST1**

**Location:** At the facial region, directly below the pupil and between the orbital inferior margin and the eyeball.

**ST2**

**Location:** At the facial region, at the site of the infraorbital foramen.

**Manipulation type:** Pressing Manipulation, as described above.

**Duration time:** 1 minute.

**Intensity:** It is advisable to experience a local sensation of soreness and distension.


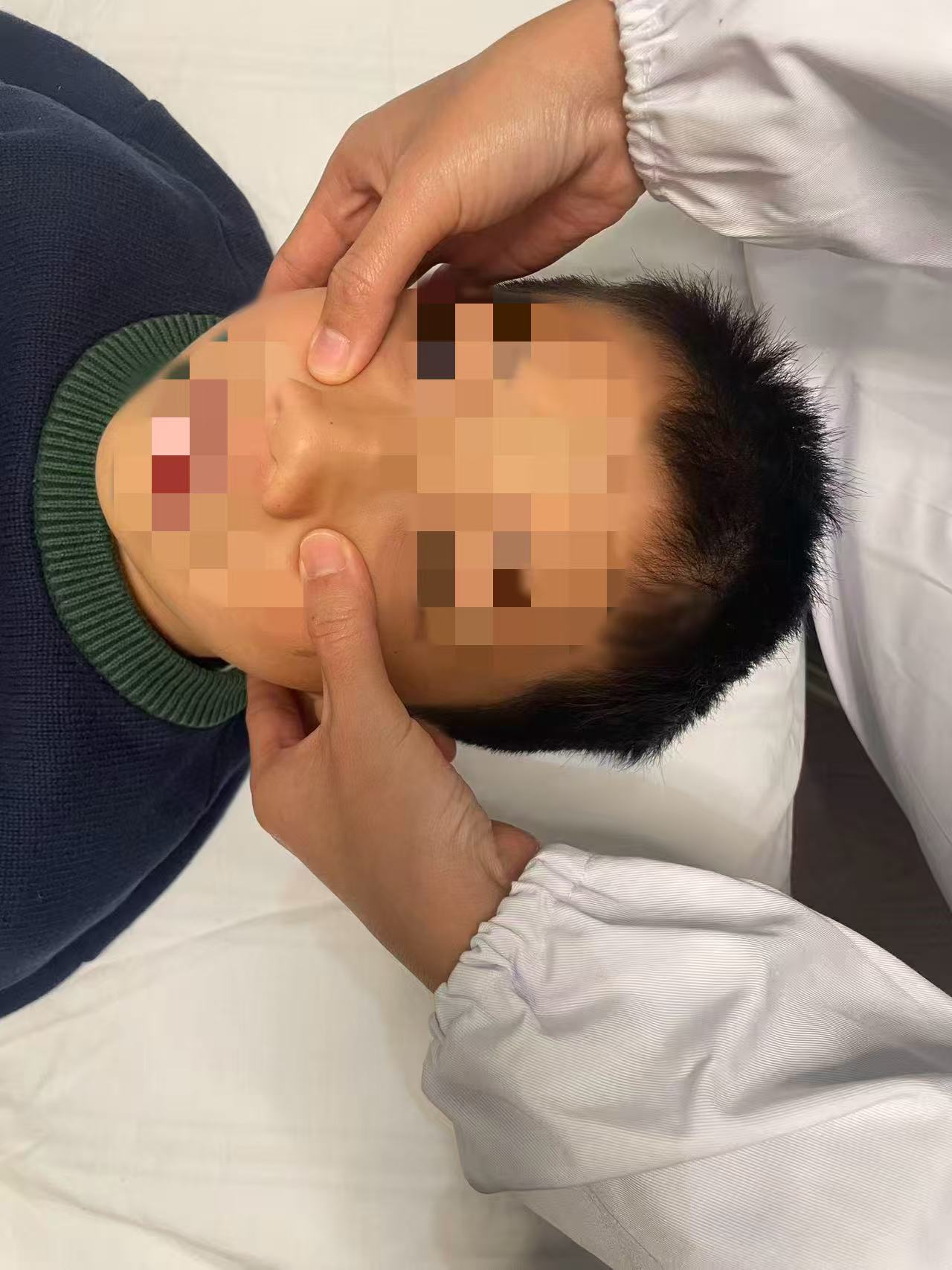

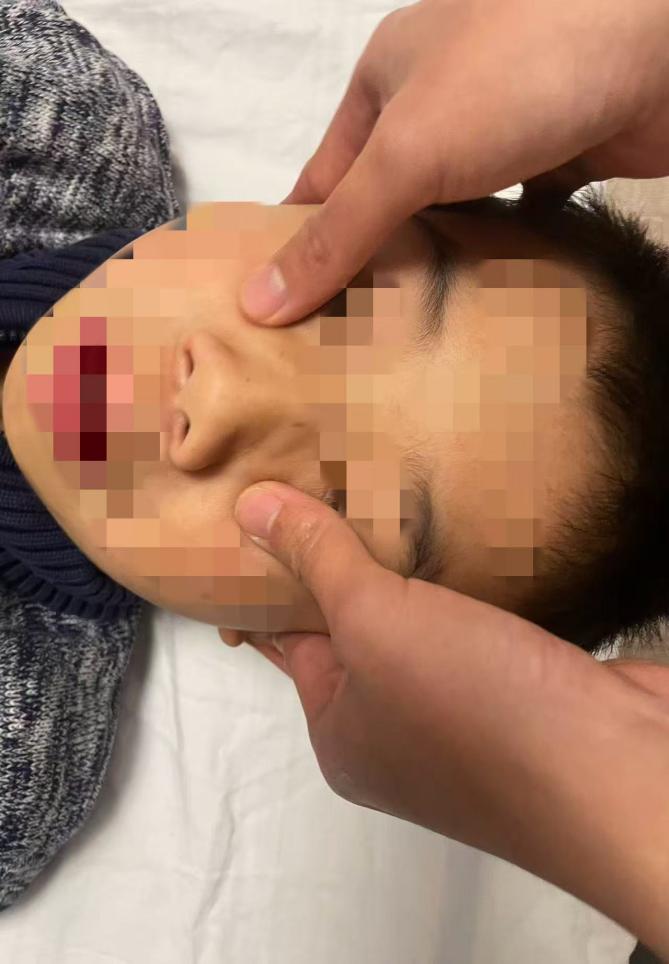


**Tianmen
Location:** The midpoint between the two eyebrows forms a straight line to the frontal hairline.

**Manipulation type:** Pushing Manipulation. Starting from below, the thumbs move upwards from the midpoint of the brow towards the hairline, alternately applying pressure.

**Duration time:** 50 times.

**Intensity:**It is advisable to ensure that the local skin of the patient does not feel pain.

**Kangong**

**Location:** From the beginning of the brow to the end of the brow forms a horizontal line.

**Manipulation type:** Pushing apart with the pads of both thumbs from the brow ridge towards the brow tail simultaneously.

**Duration time:** 50 times.

**Intensity:** It is advisable to ensure that the local skin of the patient does not feel pain.


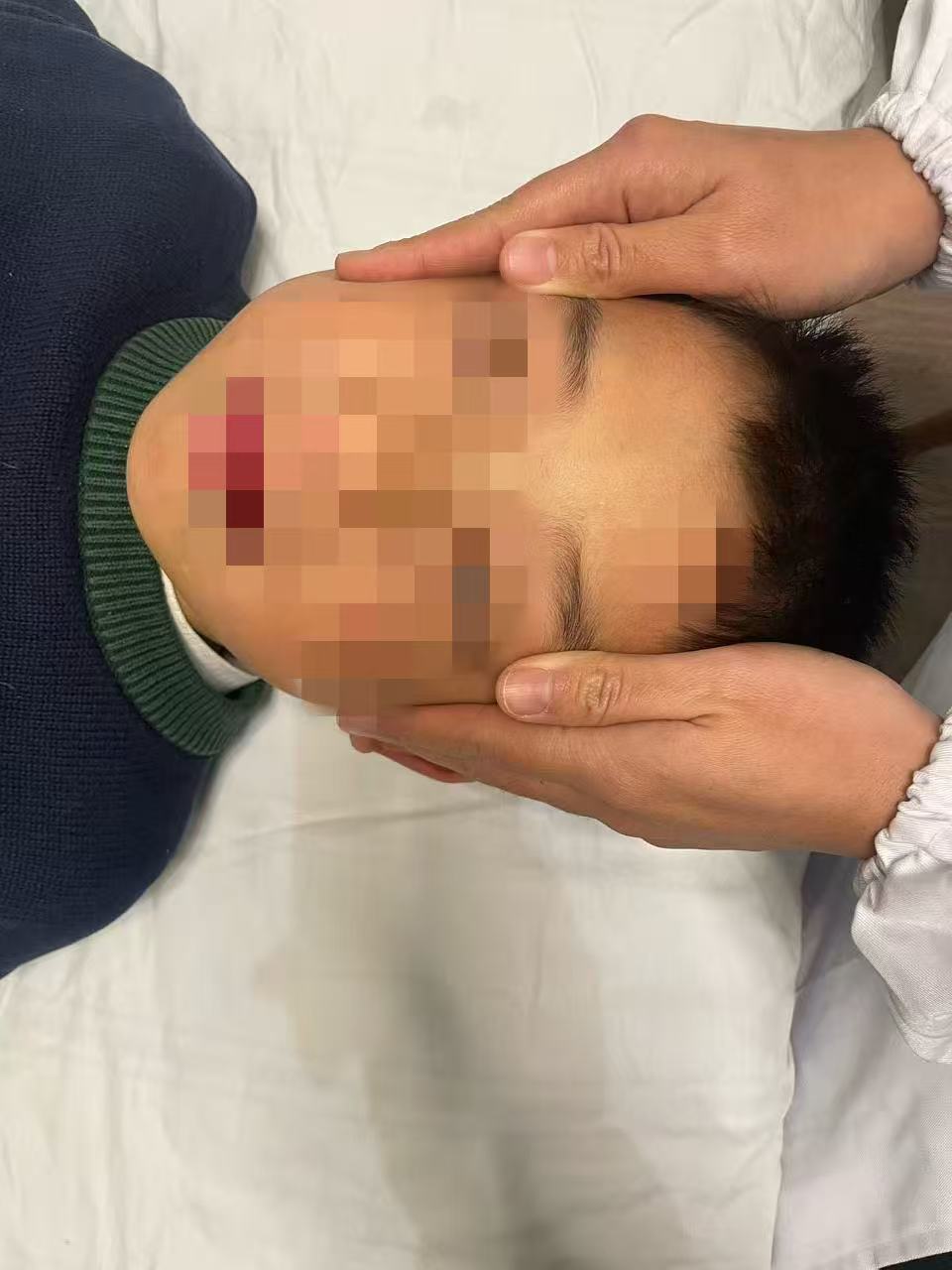

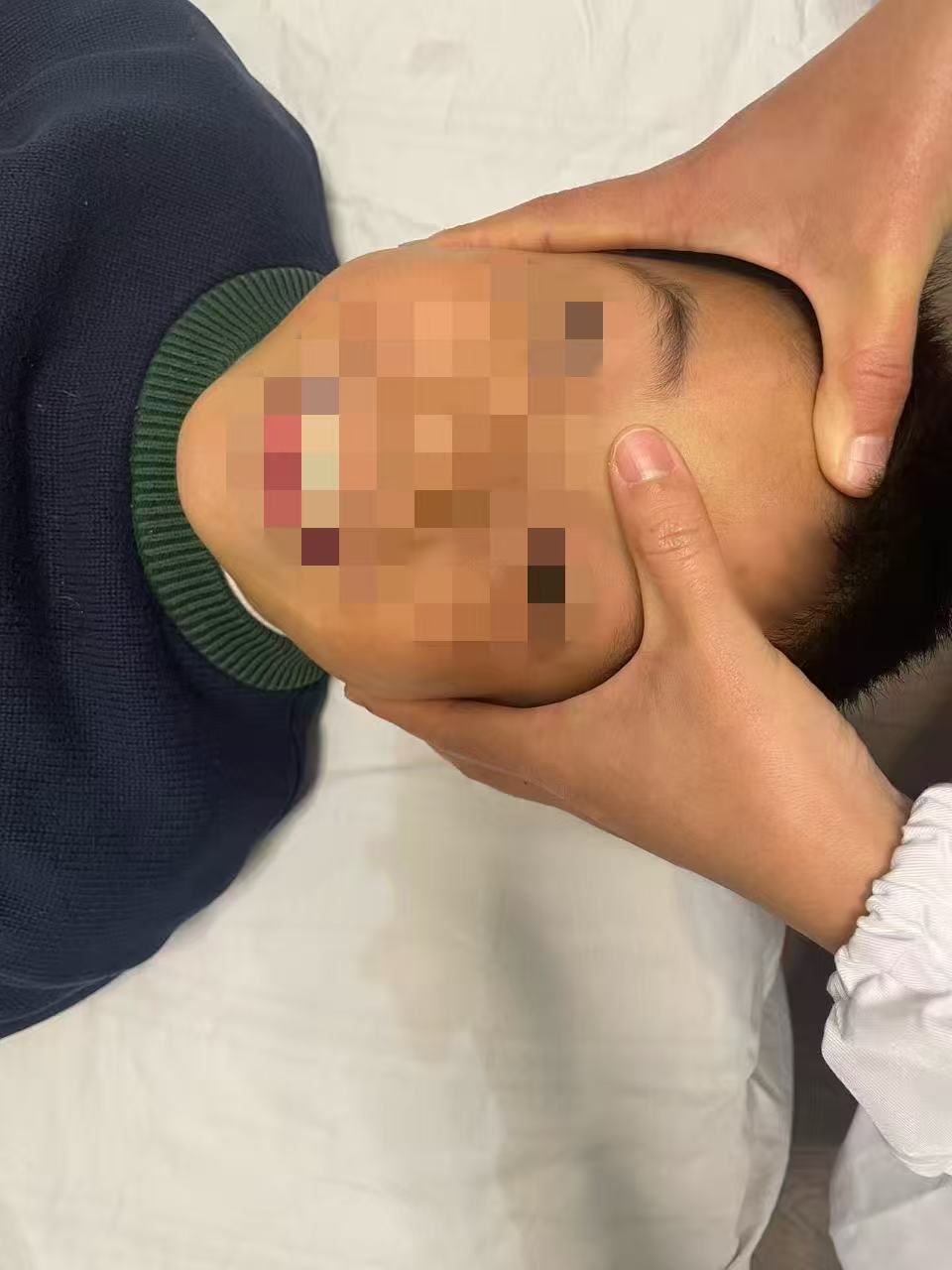


**LI20**

**Location:** At the midpoint of the lateral margin of the alar of the nose, in the nasolabial groove.

**Manipulation type:** Kneading Manipulation. As described above.

**Duration time:** 1 minute.

**Intensity:** It is advisable to ensure that the local skin of the patient does not feel pain.

**EX-HN5**

**Location:** At the depression approximately 1 cun posterior to the midpoint of the line connecting the end of the eyebrow and the outer canthus.

**Manipulation type:** Kneading Manipulation. Using the pad of the middle finger, perform circular kneading movements.

**Duration time:** 1 minute.

**Intensity:** It is advisable to ensure that the local skin of the patient does not feel pain.


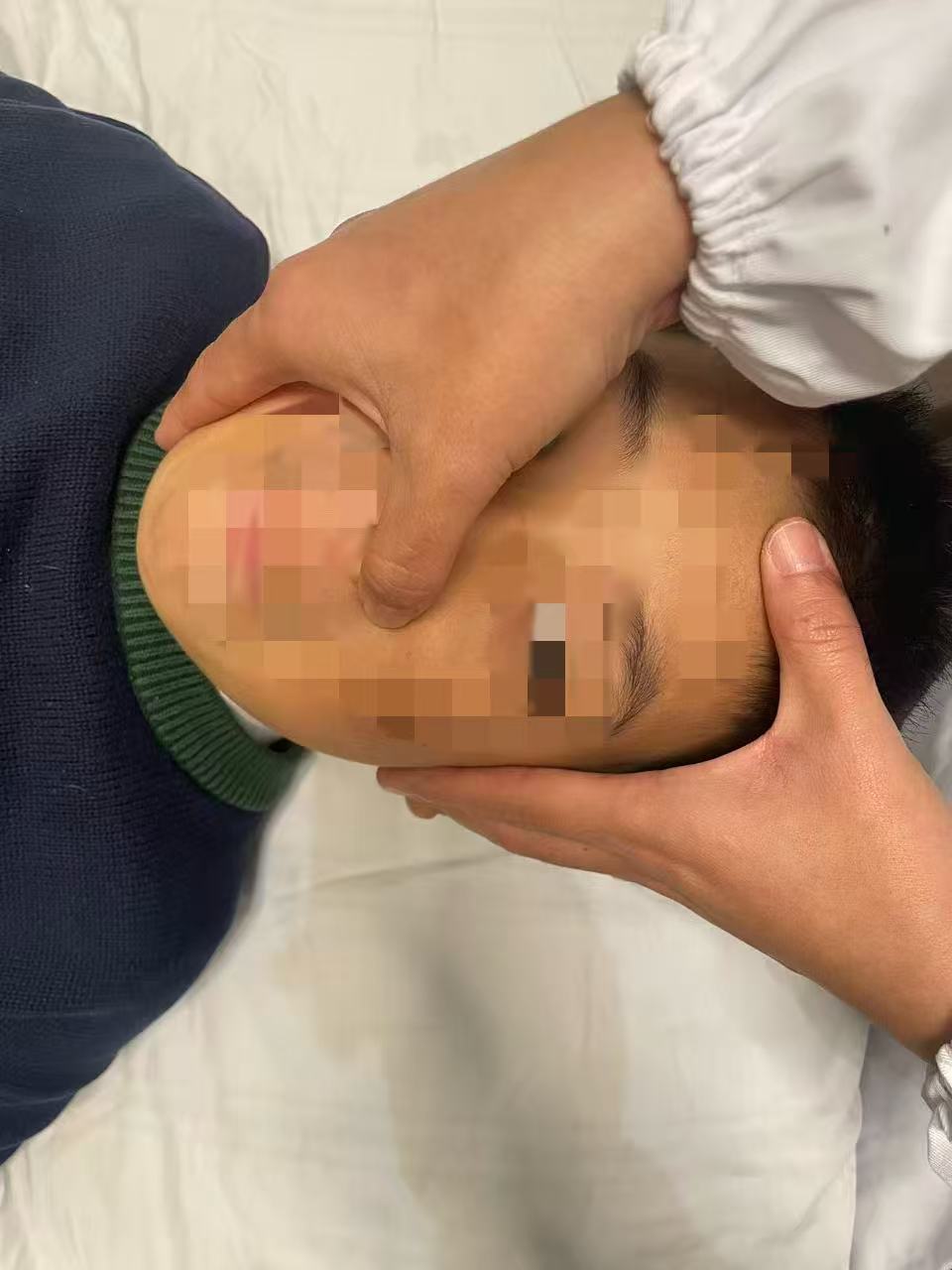

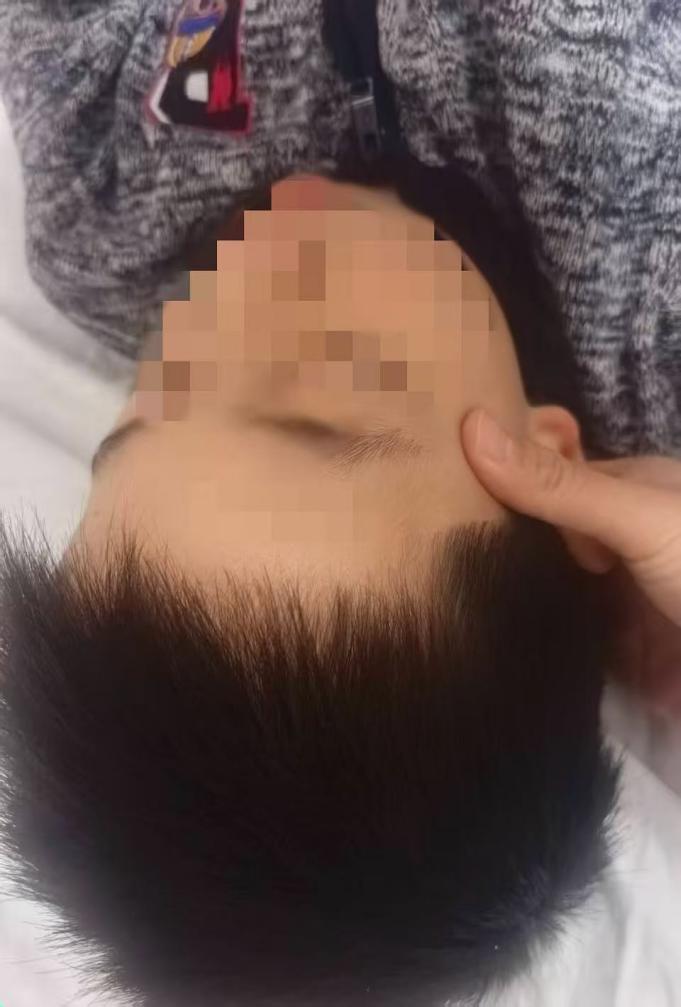


**BL23**

**Location:** At 1.5 cun lateral to the midline, inferior to the spinous process of the second lumbar vertebra.

**Manipulation type:** Rubbing manipulation. Using the palm or the thenar eminence to firmly press against the skin and perform rapid linear reciprocating rubbing movements.

**Duration time:** 1 minute.

**Intensity:** It is preferable for the local area to exhibit a slight warmth without causing any pain to the skin.

**GV4**

**Location:** At the depression below the spinous process of the second lumbar vertebra on the spinal midline.

**Manipulation type:** Rubbing manipulation. As described above.

**Duration time:** 1 minute.

**Intensity:** It is preferable for the local area to exhibit a slight warmth without causing any pain to the skin.

**BL31-34**

**Location:** At the sacral region, directly opposite the first, second, third, and fourth sacral posterior foramina, there are four acupoints on each side, totaling eight acupoints on both sides.

**Manipulation type:** Rubbing manipulation. As described above.

**Duration time:** 1 minute.

**Intensity:** It is preferable for the local area to exhibit a slight warmth without causing any pain to the skin.


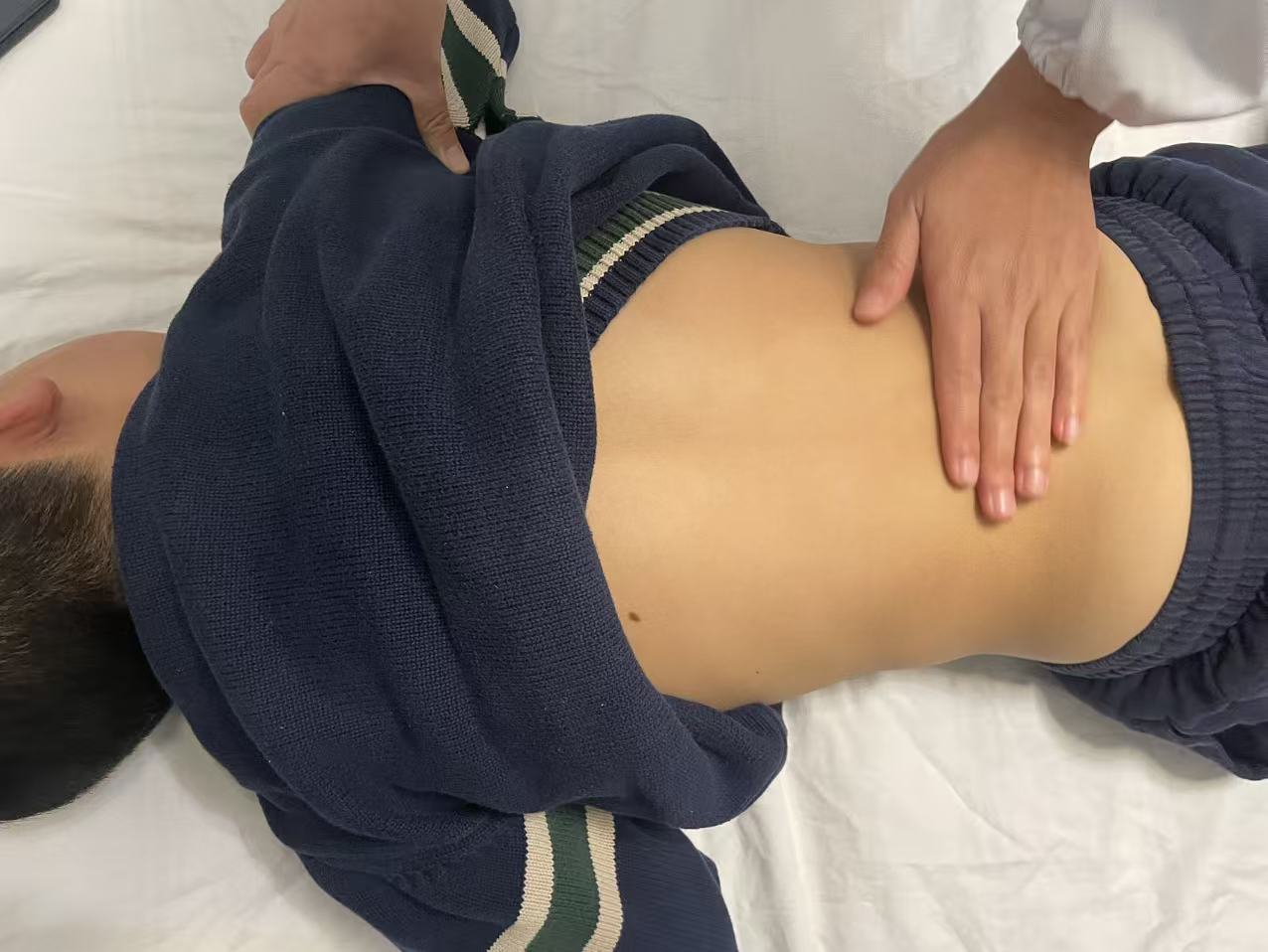


**Governor Vessel**

**Location:** Along the midline of the back, a straight line extending from the seventh cervical spinous process to the coccyx.

**Manipulation type:**

Kneading manipulation. Gently Kneading the entire spine (Governor Vessel) from top to bottom using the thumb, repeating the process three times;

Spine pinching. Using the thumb and index finger, pinch and lift the skin of the spinal column surface, performing this action from bottom to top three times in one minute.

**Duration time:** 1 minute.

**Intensity:** It is advisable to ensure that the local skin of the patient does not feel pain.


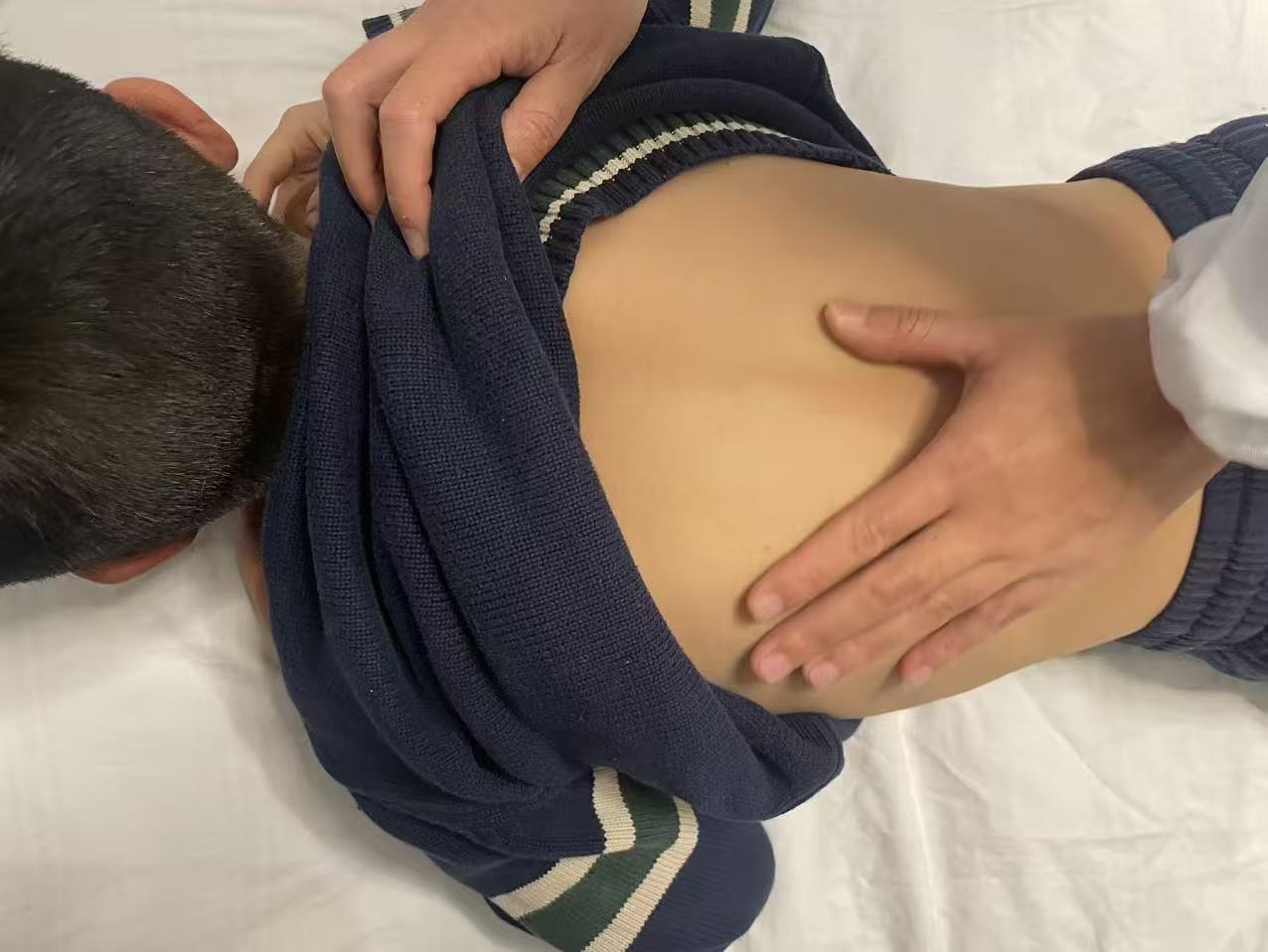

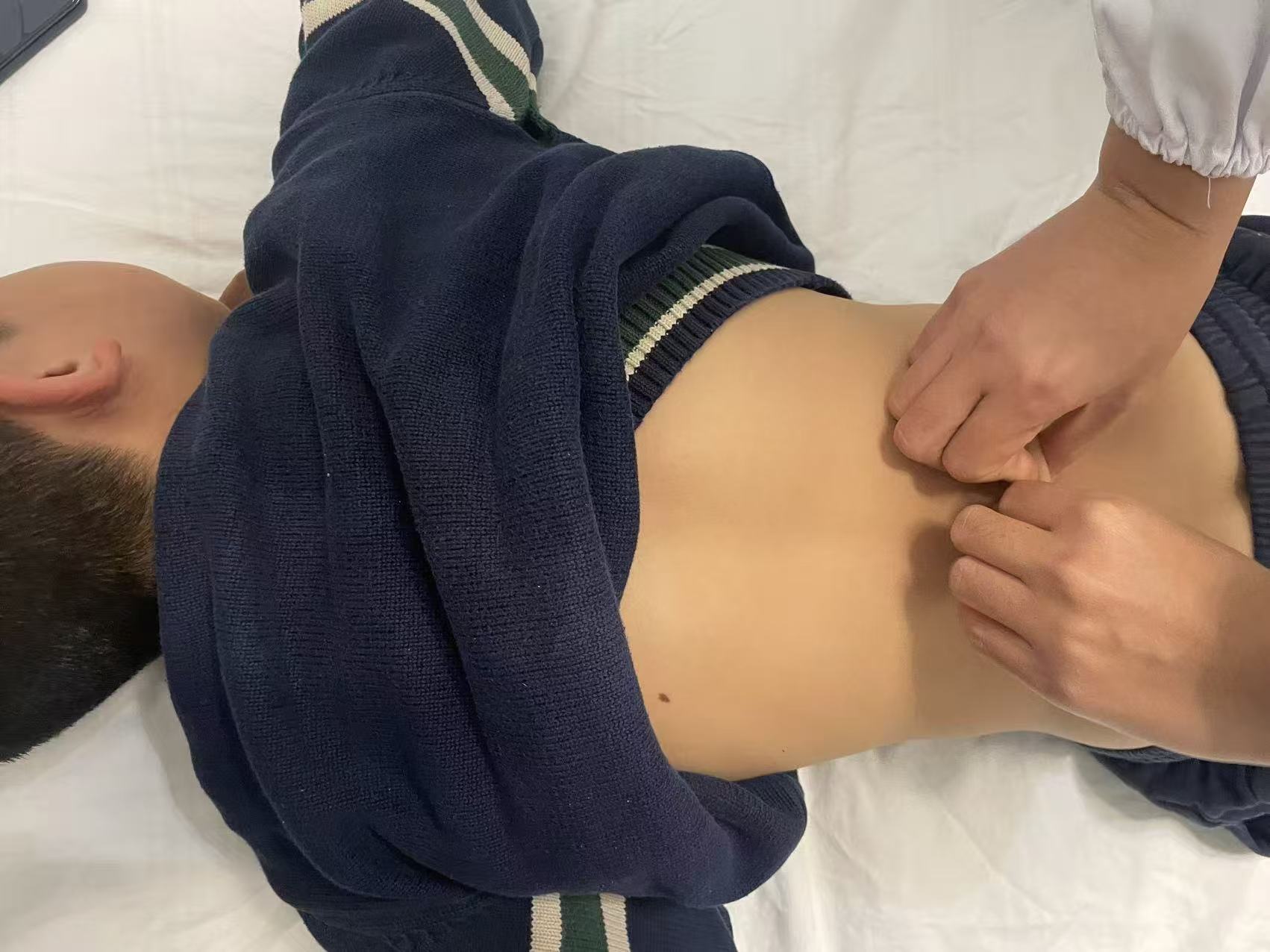

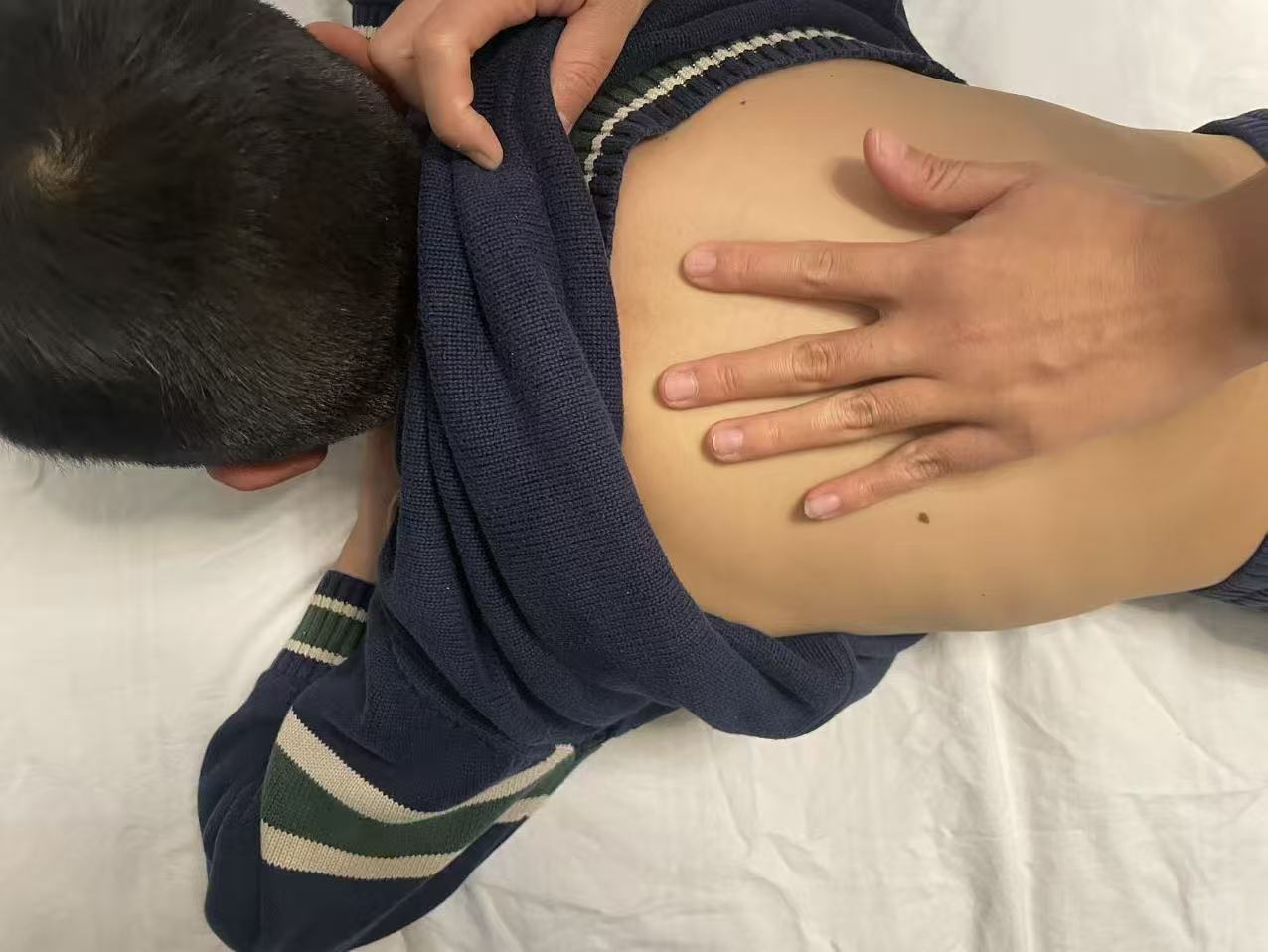


**Bladder Meridian**

**Location:** On the back area, the first lateral line is located 1.5 cun away from the spine, while the second lateral line is situated 3 cun away from the spine.

**Manipulation type:** Kneading manipulation. Kneading the first and second lateral lines of the Bladder meridian from top to bottom using the index and middle fingers, repeating the process three times.

**Intensity:** It is advisable to ensure that the local skin of the patient does not feel pain.

**GV20**

**Location:** The midpoint of the line connecting the superior points of the two ears.

**Manipulation type:** Circular rubbing manipulation. Gently rubbing the local skin with the palm or the thumb and the index, middle, and ring fingers in a ring-like manner.

**Duration time:** 1 minute.

**Intensity:** It is advisable to ensure that the local skin of the patient does not feel pain.

**GV22**

**Location:** The midpoint of the frontal hairline, 2 cun directly upward.

**Manipulation type:** Circular rubbing manipulation. As described above.

**Duration time:** 1 minute.

**Intensity:** It is advisable to ensure that the local skin of the patient does not feel pain.


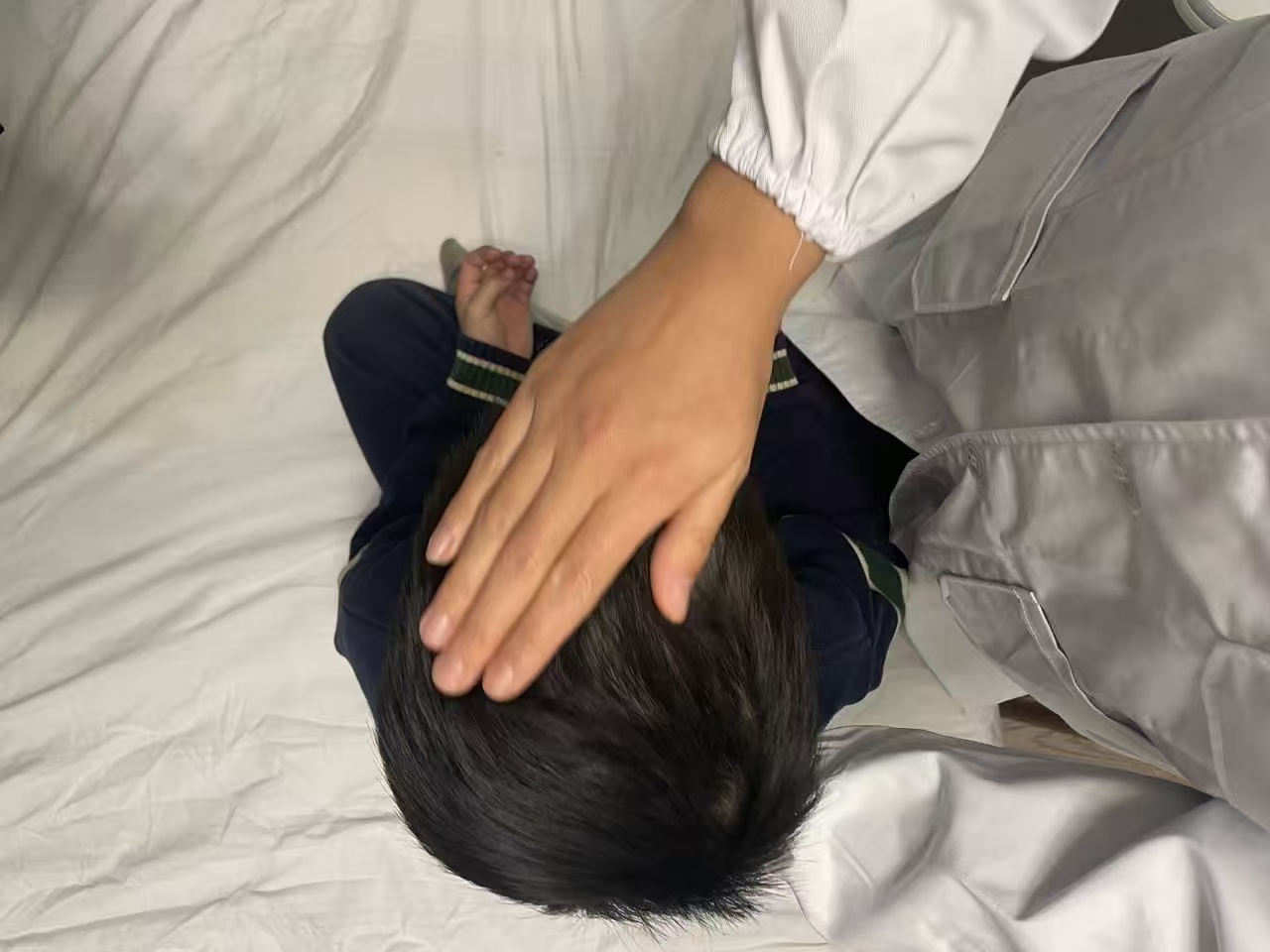

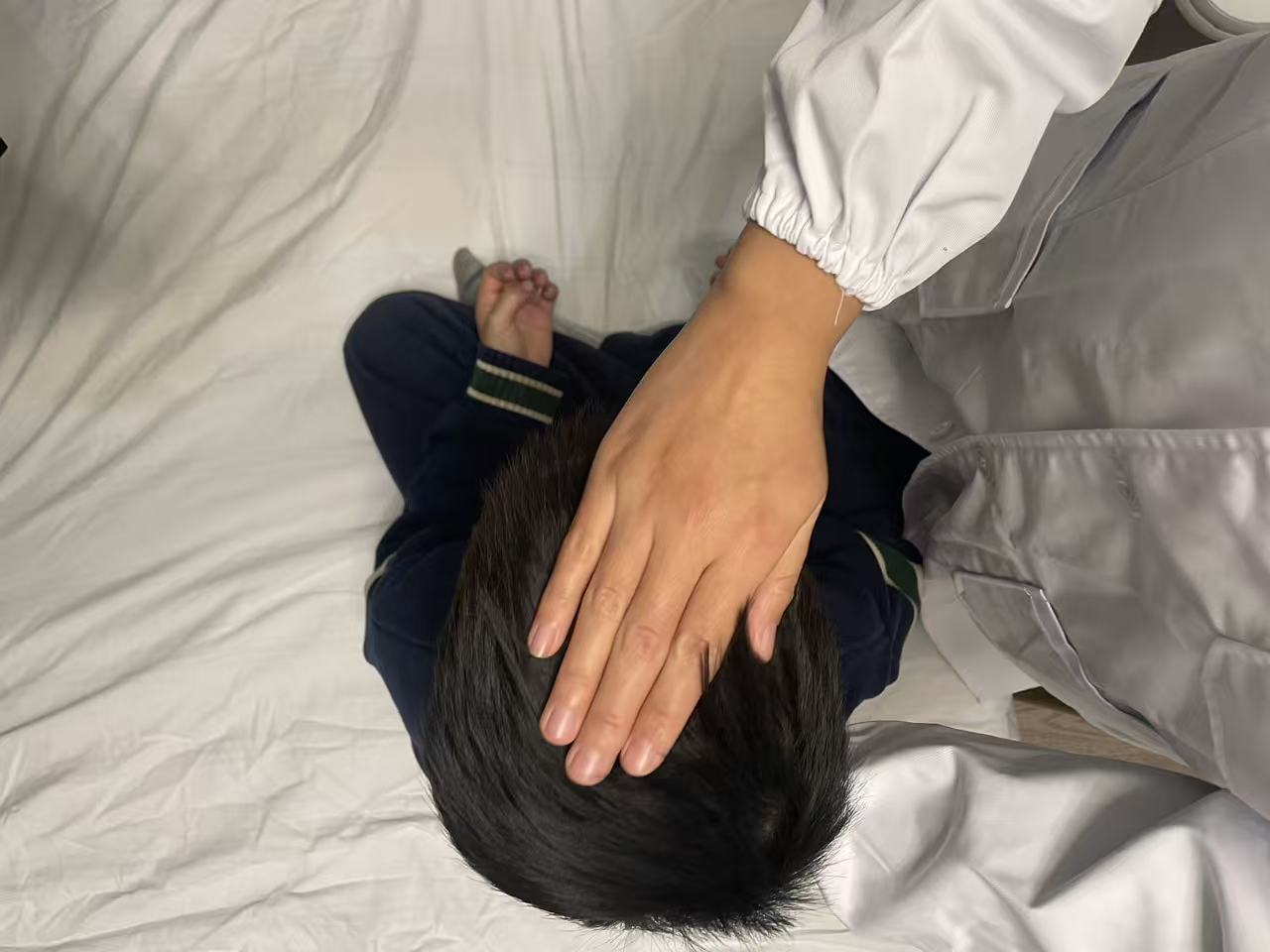

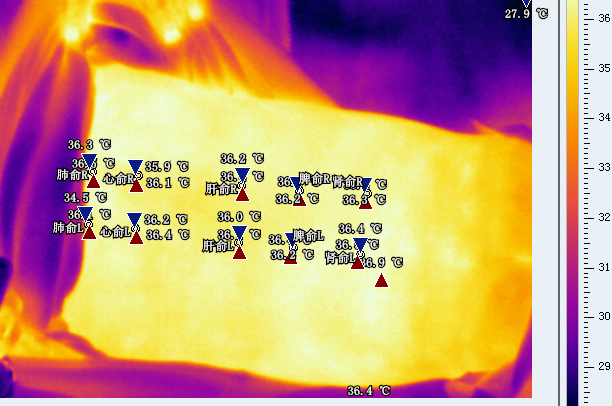

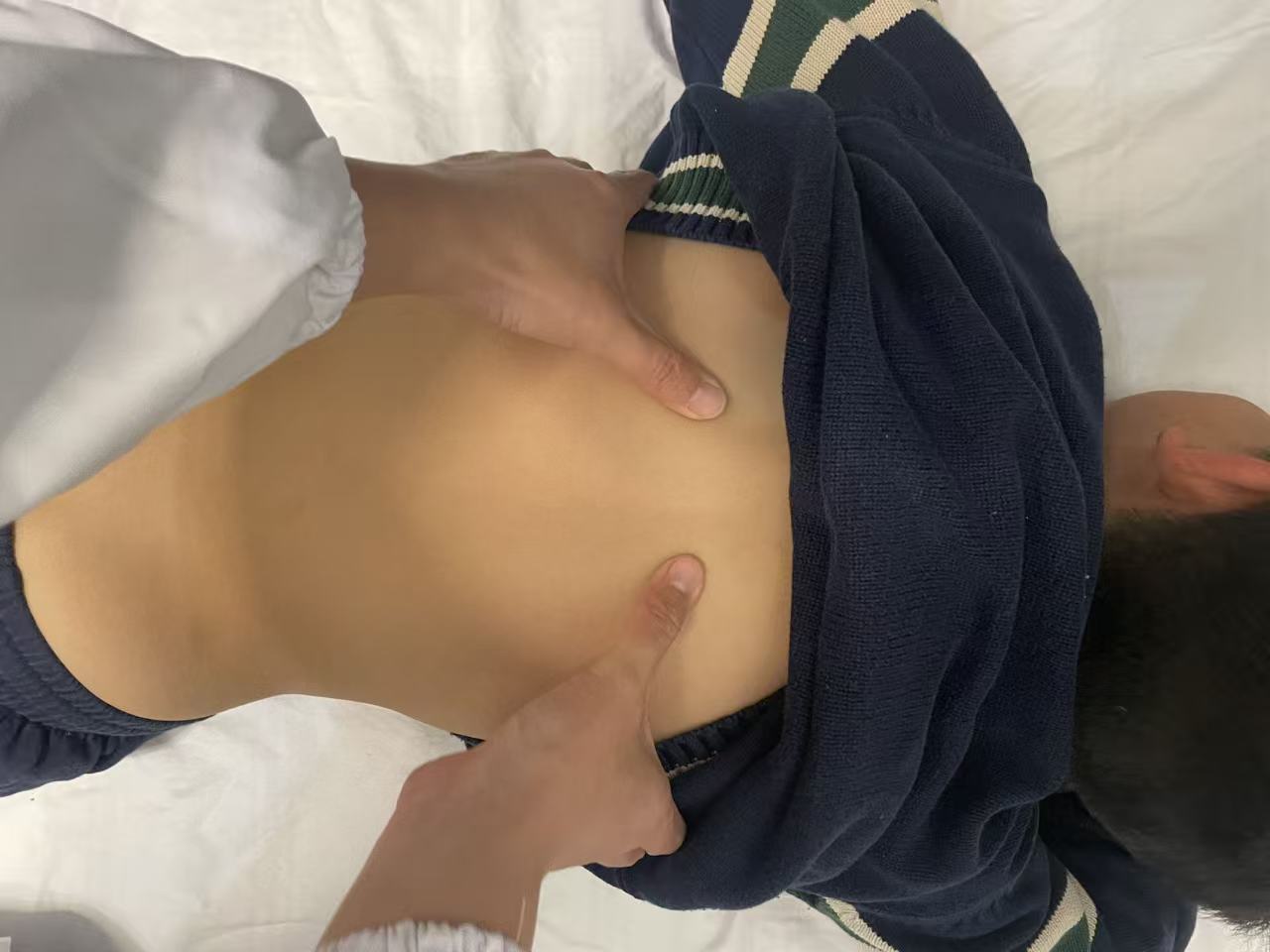


Selective stimulation of back acupoints sensitization points reveals temperature sensitization phenomena at the positions of BL18, BL23, BL13, and BL20.

**Abbreviation**

**ST:** Stomach Meridian of Foot-Yangming, ST

**TE:** Triple Energizers Meridian of Hand-Shaoyang, TE

**LI:** Large Intestine Meridian of Hand-Yangming, LI

**BL:** Gallbladder Meridian of Foot-Shaoyang, BL

**GV:** Governor Vessel, GV

**EX-HN:** Extraordinary Point of Head and Neck, EX-HN

**Cun:** The width of the interphalangeal joint of the patient's thumb is taken as 1 cun.

**1 cun**


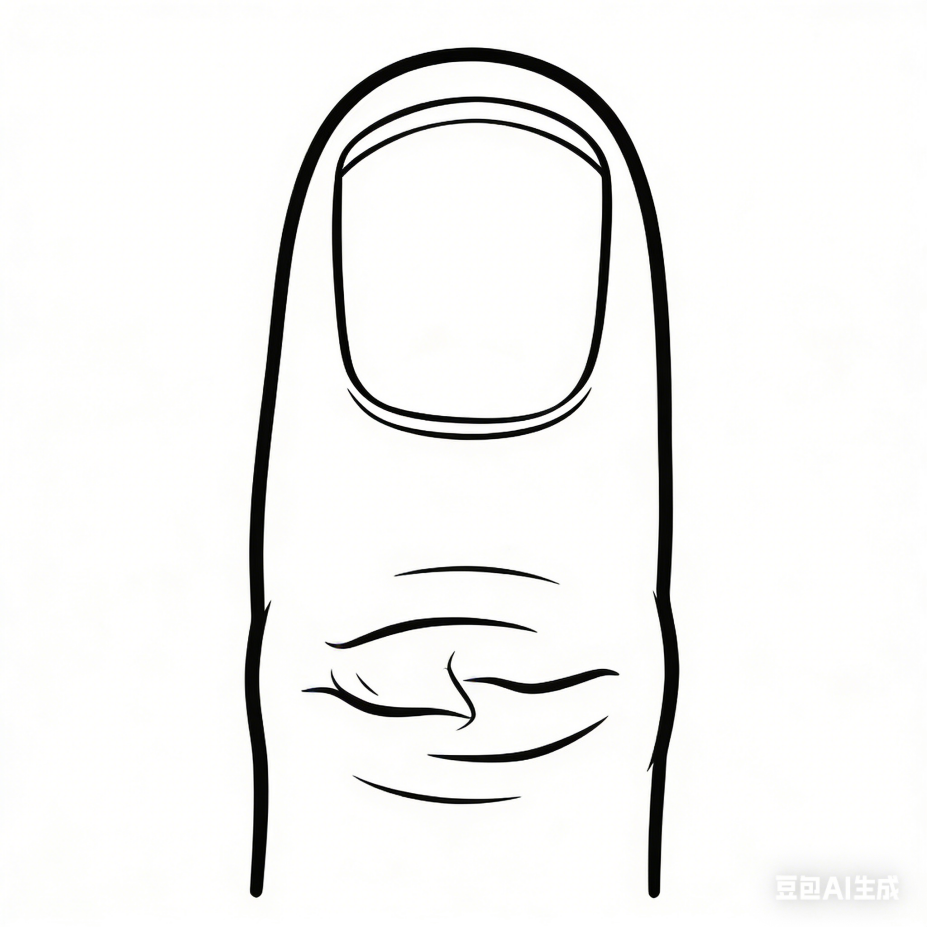


**All the locations of acupoints is be based on the WHO Standard Acupuncture Point Locations 2010 (ISBN: 9787117123327).**
