# Supplementary material for: Efficacy of Tuina for Myopia in Children: Protocol for a Randomized Controlled Trial
Source: JMIR Res Protoc. 2026 Feb 12;15:e79324. doi: 10.2196/79324 (PMC12900509; doi:10.2196/79324)

Multimedia Appendix 2

***Selective Spinal Manipulation for the Treatment of Myopia in Children***

**Clinical Observation**

**Informed Consent**

Version number of the informed consent form: 2.0 Informed Consent Form Version Date: 2024.4

Clinical trial institution: The Second Affiliated Hospital of Yunnan University of Chinese Medicine

Principal Investigator: Xiantao Tai

**Dear xxx,**

**Hello！**

**We would like to invite you to participate in a clinical study (Study Title: Randomized Controlled Clinical Trial of Selective Spinal Manipulation for the Treatment of Myopia in Children). Before deciding whether to participate in this study, please read the following information carefully. It will help you understand the study, the rationale for its conduct, the procedures and duration of the study, as well as the potential benefits, risks, and discomforts associated with participation. If you wish, you may also discuss the matter with your relatives or friends, or consult your physician for further clarification to assist in making your decision.**

**Introduction**

1. **Research Nature, Background, and Objectives of the Trial**

In recent years, numerous randomized controlled trials (RCTs) have demonstrated that spinal manipulation therapy may be an effective treatment for myopia in adolescents. However, methodological limitations have constrained the quality and reliability of these studies. Therefore, we designed a multicenter randomized controlled trial to evaluate the efficacy and safety of selective spinal manipulation in treating myopic children, and to compare the therapeutic effects of topiramate eye drops with selective spinal manipulation techniques, aiming to provide a safe and effective treatment option for adolescent myopia.

**II. Who Should Not Participate**

① patients with ocular inflammation, facial skin lesions, or infectious foci;

② Patients with comorbid severe systemic diseases, such as cardiac, cerebrova- scular, hepatic, renal, hematopoietic system, and psychiatric disorders;

③ patients with a history of congenital hereditary high myopia;

④ Concomitant pathological changes in the fundus of myopia and/or significant visual impairment or other ocular diseases that may affect the assessment of treatment efficacy.

⑤ Children with neurological and psychiatric disorders (such as palsy cerebral, autism, spina bifida, anorexia nervosa).

⑥ Myopia exceeding 600 diopters;

⑦ Under 6 years of age or over 12 years of age.

**III. What Will Be Required to Participate in the Study**

(1) Prior to enrollment in the study, physicians will inquire about and document the medical history. Your child is a qualified participant, and you may decide to participate in the study by signing the informed consent form. If you do not wish to participate in the study, we will proceed with treatment according to your preference.

(2) If you agree to participate in the study, the process will proceed as follows:

Your child will be randomly assigned to either the selective spinal *Tuina* trial group or the drug-positive control group for an 8-week treatment period (*Tuina* therapy: once every two days for 20 minutes per session; drug therapy: topiramate eye drops, once every two days for 2-3 drops per application).

Visual acuity, cycloplegic equivalent diopter, intraocular pressure (IOP), and axial length will be measured before treatment, at 4 weeks, 8 weeks, and 2 weeks after treatment completion to evaluate visual improvement. Your child will undergo the study intervention in a safe and comfortable environment (you have the right to be informed about the study intervention methods; please consult the researchers if you have any questions).

(3) Other matters requiring your cooperation

You must bring your child and relevant items such as the medical record book to the hospital for treatment or examination at the time agreed upon with the doctor.

During the testing period, your child shall not receive any non-prescribed treatments. If additional pharmacological or non-pharmacological interventions are required, please consult your physician in advance.

**IV. Potential Benefits of Participating in the Study**

(1) There is potential for improvement in your condition, and this study may facilitate the application of this method to more children with myopia.

(2) You will receive quality medical services during the study period.

(3) All participants who complete the follow-up will receive 5 free *Tuina* treatments. Those who fail to complete the entire trial process will not be eligible for the free *Tuina* treatments.

**V. Potential Adverse Reactions, Risks, and Inconveniences or Discomforts Associated With Participating in the Study**

(1) Potential Adverse Reactions and Risks

Adverse reactions such as photophobia and dry eyes may occur with toperamide eye drops, which can be resolved after discontinuation of the medication.

Inappropriate Tuina pressure may lead to symptoms such as skin congestion and pain, which can be alleviated by cold compress with a towel and rest.

(2) Potential inconveniences

During the study period, you are required to attend hospital follow-up appointments on schedule and undergo vision-related examinations. This may cause you trouble or inconvenience.

Furthermore, any treatment may fail, and the condition may continue to progress due to treatment failure or the presence of comorbidities. This represents a therapeutic risk that every patient will encounter, even if they do not participate in this clinical study.

**VI. Relevant Expenses**

This study is completely free of charge. The hospital will cover the costs of all research-related examinations conducted during your participation, as well as the registration fee for follow-up visits. Additionally, the study medication will be provided free of charge. Upon completion of the study, you will receive 5 complimentary pediatric *Tuina* services. If you are also undergoing treatment and tests for other diseases, these will not be covered by the free service.

**Ⅶ. Are Personal Information Confidential?**

Yes, we will take a series of regulations to protect your personal information security.

1. Privacy Protection Measures: During the clinical trial, your personal information is subject to strict confidentiality measures. This information is stored in a secure database and can only be accessed by authorized personnel. After the trial concludes, this information must remain confidential to prevent disclosure to unauthorized individuals.

② Ethical Review and Informed Consent: The clinical trial protocol may only be implemented after obtaining approval from the ethics committee. Participants must voluntarily enroll in the clinical trial, with full disclosure of risks and benefits, and sign an informed consent form. Participants have the right to withdraw from the study at any stage without any reason, and their normal medical services and rights will not be affected in any way.

③ Data recording and preservation: All paper or electronic data from clinical trials will be properly recorded. Fees are prohibited: Medical institutions or investigators are strictly prohibited from charging participants or study subjects any fees related to the study in violation of regulations.

**Ⅷ**. **How to Obtain More Information?**

You may ask any questions regarding this study at any time. Your physician will provide you with their telephone number to address your inquiries. If you have any concerns about participating in the study, please contact the Ethics Committee Office (phone number: 0871-65954492).

If any significant new information during the study that may affect your willingness to continue participating in the study is identified, your physician will promptly notify you.

**IX. Participation in the Study is Voluntary and Withdrawal is Permitted at Any Time**

Participation in this study is entirely voluntary. You may decline to participate or withdraw from the study at any time during the research process, which will not affect your relationship with the physician, nor will it result in any loss of medical or other benefits.

your physician or investigator may discontinue your participation in this study at any time for the best interest of you.

If you do not participate in this study or withdraw from it, there are many alternative treatment options available, such as traditional Chinese medicine formulations or surgical interventions. You are not required to participate in this study solely for the purpose of treating your condition. If you withdraw from the study for any reason, you may be asked to provide information regarding your use of investigational drugs. If deemed necessary by your physician, you may also be required to undergo laboratory tests and physical examinations. These measures are highly beneficial for safeguarding your health

**Ⅹ. What Should We Do Now?**

Your participation in this study is at your own discretion. You may discuss the decision with your family or friends before making a choice. Before making the decision to participate in this study, please consult your physician regarding any questions you may have until you have fully understood the study.

Thank you for reading the above materials. If you decide to participate in this study, please inform your physician or research assistant, who will arrange all matters related to the study for you.

Please keep this information.

Informed Consent · Consent Signature Page

**I have read the above-mentioned introduction to this study and had the opportunity to discuss and ask questions about this study with the physician. All my questions were satisfactorily answered.**

**I am aware of the potential risks and benefits associated with participation in this study. I acknowledge that participation is voluntary, have had sufficient time to consider it, and understand that:**

**I can always consult a doctor for more information.**

**·**

**I can withdraw from this study at any time without discrimination or retaliation, and**

**·**

**my medical treatment and rights will not be affected.**

**I am also fully aware that if I withdraw from the study midway, particularly due to medication- related reasons, informing the physician of the disease progression and completing the corresponding physical and biochemical examinations would be highly beneficial for both myself and the entire study.**

**If I need any other medication due to changes in my condition, I will consult my doctor in advance or inform them truthfully afterwards.**

**I consent to the review of my research materials by the drug regulatory authority, ethics committee, or sponsor representatives.**

**I agree□ or refuse□ Other studies, in addition to this one, have used my medical records and pathological specimens.**

**I will receive a copy of the informed consent form signed and dated. Finally, I decided to consent to participate in this study.**

**Subject signature: _ or signature of the guardian__ (How many age groups require dual signatures)**

**Patient contact number: date：**


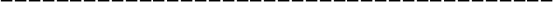


**I confirm that the patient has been fully informed of the details of the trial, including his/her rights, potential benefits and risks, and provided with a copy of the signed informed consent form.**

**Researcher's signature: Date:**

**Researcher's work phone: cell-phone number：**


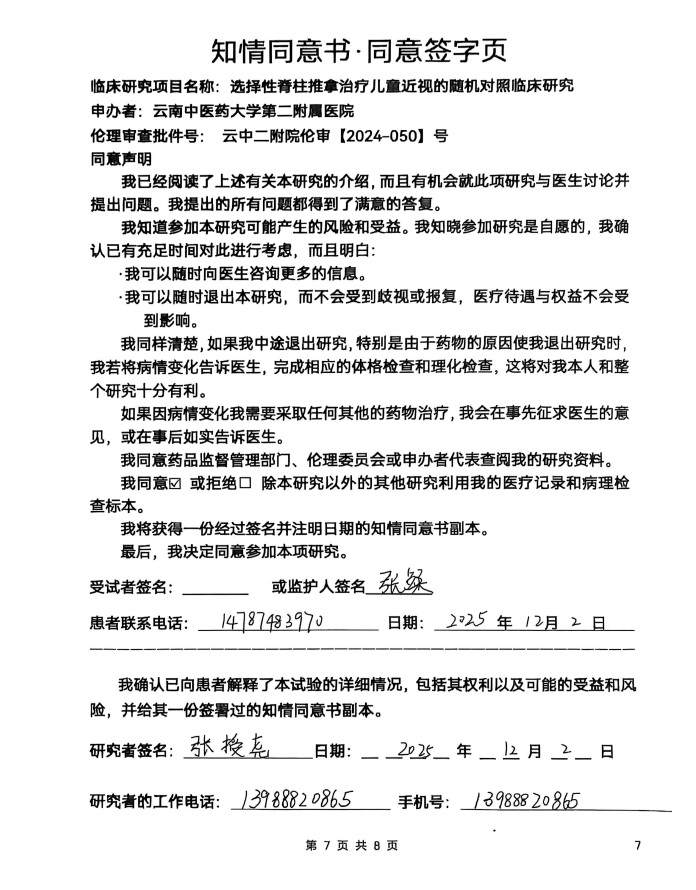

Supplement: Multimedia Appendix 2 [file resprot-v15-e79324-s002.docx]
